# Zearalenone disturbs the reproductive-immune axis in pigs: the role of gut microbial metabolites

**DOI:** 10.1186/s40168-022-01397-7

**Published:** 2022-12-19

**Authors:** Shujin Wang, Wei Fu, Xueya Zhao, Xiaojiao Chang, Hujun Liu, Lin Zhou, Jian Li, Rui Cheng, Xin Wu, Xi Li, Changpo Sun

**Affiliations:** 1 Institute of Life Sciences, Chongqing Medical University, Chongqing, 400032 The People’s Republic of China; 2 Tianjin Institute of Industrial Biotechnology, Chinese Academy of Sciences, Tianjin, 300308 The People’s Republic of China; 3 Key Laboratory of Qinghai-Tibetan Plateau Animal Genetic Resource Reservation and Utilization, Ministry of Education, Southwest Minzu University, Chengdu, 610000 The People’s Republic of China; 4 Academy of National Food and Strategic Reserves Administration, Beijing, 100037 The People’s Republic of China; 5 Shenzhen Premix INVE Nutrition, Co., LTD., Shenzhen, 518100 The People’s Republic of China; 6 CAS Key Laboratory of Agro-ecological Processes in Subtropical Region, Institute of Subtropical Agriculture, Chinese Academy of Sciences, Changsha, 410125 The People’s Republic of China; 7 Standards and Quality Center of National Food and Strategic Reserves Administration, Beijing, 100037 The People’s Republic of China

**Keywords:** Mycotoxin, Mycoestrogen, Zearalenone, Butyrate, Lipopolysaccharides, Immunity, Reproductive system, Immune system, Gastrointestinal tract, *Sus scrofa*

## Abstract

**Background:**

Exposure to zearalenone (ZEN, a widespread *Fusarium* mycotoxin) causes reproductive toxicity and immunotoxicity in farm animals, and it then poses potential threats to human health through the food chain. A systematic understanding of underlying mechanisms on mycotoxin-induced toxicity is necessary for overcoming potential threats to farm animals and humans. The gastrointestinal tract is a first-line defense against harmful mycotoxins; however, it remains unknown whether mycotoxin (e.g., ZEN)-induced toxicity on the reproductive-immune axis is linked to altered gut microbial metabolites. In this study, using pigs (during the three phases) as an important large animal model, we investigated whether ZEN-induced toxicity on immune defense in the reproductive-immune axis was involved in altered gut microbial-derived metabolites. Moreover, we observed whether the regulation of gut microbial-derived metabolites through engineering ZEN-degrading enzymes counteracted ZEN-induced toxicity on the gut-reproductive-immune axis.

**Results:**

Here, we showed ZEN exposure impaired immune defense in the reproductive-immune axis of pigs during phase 1/2. This impairment was accompanied by altered gut microbial-derived metabolites [e.g., decreased butyrate production, and increased lipopolysaccharides (LPS) production]. Reduction of butyrate production impaired the intestinal barrier via a GPR109A-dependent manner, and together with increased LPS in plasma then aggravated the systemic inflammation, thus directly and/or indirectly disturbing immune defense in the reproductive-immune axis. To validate these findings, we further generated recombinant *Bacillus subtilis* 168-expressing ZEN-degrading enzyme *ZLHY-6* (the Bs-Z6 strain) as a tool to test the feasibility of enzymatic removal of ZEN from mycotoxin-contaminated food. Notably, modified gut microbial metabolites (e.g., butyrate, LPS) through the recombinant Bs-Z6 strain counteracted ZEN-induced toxicity on the intestinal barrier, thus enhancing immune defense in the reproductive-immune axis of pigs during phase-3. Also, butyrate supplementation restored ZEN-induced abnormalities in the porcine small intestinal epithelial cell.

**Conclusions:**

Altogether, these results highlight the role of gut microbial-derived metabolites in ZEN-induced toxicity on the gut-reproductive-immune axis. Importantly, targeting these gut microbial-derived metabolites opens a new window for novel preventative strategies or therapeutic interventions for mycotoxicosis associated to ZEN.

**Supplementary Information:**

The online version contains supplementary material available at 10.1186/s40168-022-01397-7.

## Highlights

I. Zearalenone directly and/or indirectly disturbs immune defense in the reproductive-immune axis;

II. Zearalenone-induced toxicity on the reproductive-immune axis is accompanied by systemic inflammation caused by altered gut microbial metabolites (e.g., decreased butyrate, and increased lipopolysaccharides);

III. Targeting these gut microbial metabolites counteracts Zearalenone-induced toxicity on the gut-reproductive-immune axis.

Video Abstract

**Supplementary Information:**

The online version contains supplementary material available at 10.1186/s40168-022-01397-7.

## Introduction

Fungal growth and mycotoxin contamination are major concerns facing agricultural production worldwide. Contamination with *Fusarium* mycotoxins was found to affect 84% of agricultural commodity samples globally in a 2015 survey of over 8000 samples [1]. Zearalenone (ZEN) is a predominant *Fusarium* mycotoxin, but also produced by other fungal genera, and frequently co-occurs with deoxynivalenol on a worldwide basis in cereal grains, animal feeds, and forages [2]. As it is difficult to remove completely during the manufacturing process, ZEN affects the entire human food chain through a range of food products [3, 4]. It not only causes significant economic losses in crops and farm animals but also poses potential threats to human health through the food chain [5, 6].

The European Food Safety Authority (ESFA) firstly established the daily exposure limit for ZEN as 250 ppb in complementary and complete feeding stuffs for sows and fattening pigs (European Commission, 2006) and 100 ppb in human consumption (European Commission, 2007), respectively [5]. According to the regulations of the General Administration of Quality Supervision, Inspection and Quarantine of China (AQSIQ) and Standardization Administration of China (SAC), the maximal level of ZEN allowed in animal feed is 500 ppb (AQSIQ and SAC, 2017). Of note, exposure to excessive mycotoxin causes serious health problems for farm animals, including reproductive and developmental toxicity, carcinogenic, hepatotoxic, nephrotoxicity, and immunotoxicity [6–9]. Once ingested by farm animals, ZEN and its metabolites can be detected in most tissues such as liver, kidney, and reproductive organs and its product (e.g., meat and milk) [2]. Humans may be indirectly exposed to ZEN through the consumption of these tissues and its products [10, 11], and ZEN-induced toxicity then contributes to precocious puberty, hyperplastic, and neoplastic endometrium, cervical, ovarian, breast cancer, and genotoxicity [12–15]. Therefore, a systematic understanding of the underlying mechanisms of mycotoxin (e.g., ZEN)-induced toxicity is necessary for overcoming potential threats to farm animals and human health.

ZEN is mainly metabolized and accumulated in immune and reproductive organs. Thus, these organs are potential key target organs for this mycotoxin and/or its metabolites to exert its toxic effects [16–19]. However, mycotoxins are introduced into the organism from food, they first come to interact with the gastrointestinal tract that functions as the first barrier against harmful mycotoxins [20]. Thus, the potential damage of mycotoxins to the gastrointestinal tract cannot be ignored [21]. The gastrointestinal tract is mainly where gut microbiota resides: it is known for its role in modulating the immune system, endocrine system, and digestive processes [21–23]. Notably, gut microbiota has a high capacity to degrade mycotoxins toxicity by inhibiting the effects of mycotoxins on the major functional proteins and small peptides at the cellular level [24], yet the underlying mechanism of mycotoxins on intestinal barrier dysfunction has remained unknown. Until now, moreover, no systematic in vivo studies have been presented to identify whether mycotoxin-induced toxicity in the reproductive-immune axis is accompanied by altered gut microbial-derived metabolites.

Domesticated pigs are a major food source worldwide and thus important in global food security, but large-scale pig farming faces challenges of mycotoxin-contaminated food [25]. Increasing the understanding of the interactions among the gastrointestinal tract, reproductive system, and immune system has the potential to facilitate knowledge-based development of sustainable pig production by increasing feed efficiency and general feed health in pig farming. Also, the high similarity of pigs to humans in anatomical size and structure, physiology, immunology, and genome enhances their potential as models for humans [26, 27]. Accordingly, we have generated pigs as an important large animal model in this study.

And, we then investigate whether ZEN-induced toxicity on the gut-reproductive-immune axis in pigs, during the three phases, is accompanied by altered gut microbial-derived metabolites. Herein, we illuminate that ZEN directly and/or indirectly disturbs immune defense in the gut-reproductive-immune axis via the systemic inflammation, which is involved in altered gut microbial-derived metabolites [e.g., decreased butyrate production, and increased lipopolysaccharides (LPS) production]. Notably, the regulation of gut microbial-derived metabolites, which is achieved by recombinant *Bacillus subtilis* (*B. subtilis*) 168-expressing ZEN-degrading enzyme *ZLHY-6*, counteract ZEN-induced toxicity on intestinal barrier, thus enhancing immune defense in the reproductive-immune axis.

## Results

### ZEN disturbs reproductive and immune systems

**Fig. 1:**
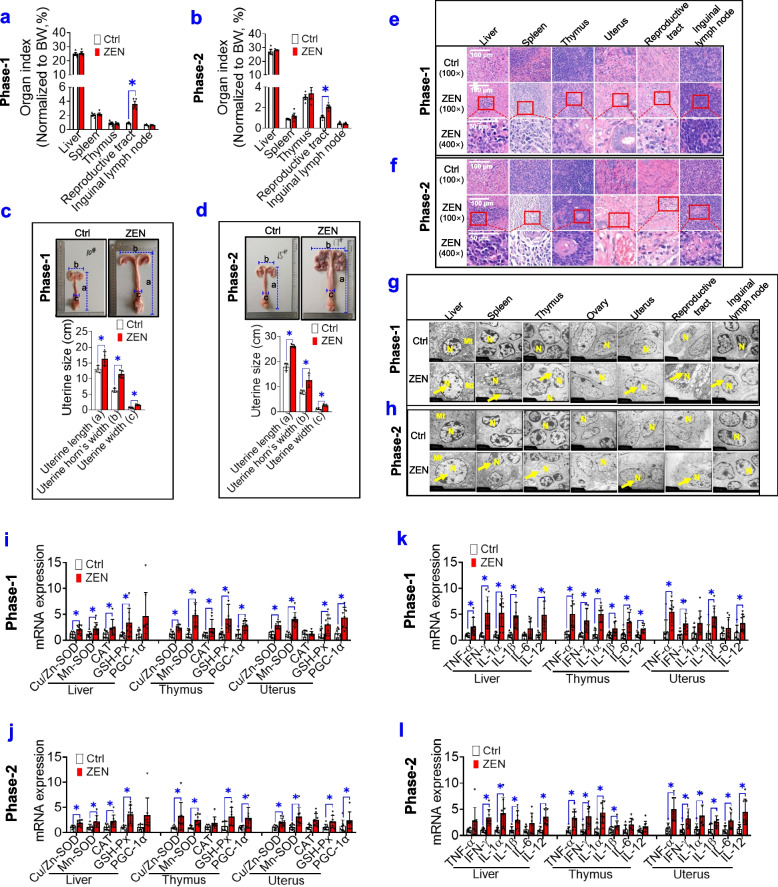
ZEN-contaminated food disturbs reproductive and immune systems. **a**, **b** The organ’s index of pigs, during phase-1 (**a**) and phase-2 (**b**), between the Ctrl group and zearalenone (ZEN) group (*n*=4). **c**, **d** The uterine size (uterine length, uterine width, and uterine horn’s width) of pigs (*n*=4). **e**, **f** Representative H&E staining of immune organs (liver, spleen, thymus, and inguinal lymph nodes) and reproductive organs (uterus and reproductive tract) from each group under ×40 magnification. The scale bar represents 100 μm. **g**, **h** Representative transmission electron microscopy (TEM) images of immune organs and reproductive organs from each group (The yellow arrows show swelling nucleus). The scale bar represents 2 μm. **i**–**l** The mRNA expression of oxidative stress markers (**i**, **j**) and pro-inflammatory cytokines (**k**, **l**) in immune organs (e.g., liver and thymus) and reproductive organs (e.g., uterus) (*n*=8). Bar values are means ± SEM. *P < 0.05

To test the toxicity effects of ZEN on both pre-starter (phase-1) and starter (phase-2) pigs, ZEN/its metabolites residues, growth performance, organ’s index, and vulvar areas were measured. Using the HPLC-MS/MS assay, we observed both phase-1 and phase-2 pigs that were exposed to ZEN displayed a higher residue of ZEN/its metabolites in all five gut sections (e.g., duodenum, jejunum, ileum, caecum, and colon), blood, immune system (e.g., liver, spleen, thymus, and inguinal lymph nodes), and reproductive system (e.g., uterus and ovary) (*P* < 0.05) (Supplemental Table S1). However, no obvious changes in growth performance were observed in pigs between the Ctrl group and ZEN group (Supplemental Table S2). Although no significant differences were found in the relative weight of immune organs in pigs between the Ctrl group and ZEN group, the reproductive tract of pigs that were exposed to ZEN showed a higher relative weight (*P* < 0.05) (Fig. 1a, b). Next, a larger vulvar area in pigs exposed to ZEN was started on day 7 (*P* < 0.05) and then on day 14 (*P* < 0.01) (Supplemental Fig. S1a-b). Also, an obvious increase in uterine size (e.g., uterine length, uterine width, and uterine horn’s width) was observed in pigs exposed to ZEN (Fig. 1c, d). Accordingly, compared with other organs, the reproductive system in pigs is a comparatively high sensitivity to ZEN.

Next, we explored whether ZEN-induced toxicity on the reproductive system was accompanied by morphologic changes, mitochondrial dysfunction, oxidative stress, and inflammation in immune organs. As indicated in Fig. 1e, f, obvious pathomorphism in reproductive and immune systems from pigs that were exposed to ZEN resulted in inflammatory lesions. Also, by using the TEM method, we observed pigs in the ZEN group showed a remarkable swelling nucleus (Fig. 1g, h). Furthermore, ZEN-induced toxicity rapidly contributed to adverse oxidative stress (e.g., increased mRNA expression of *Cu/Zn-SOD*, *Mn-SOD*, *GSH-Px*, and *PGC-1α*) (Fig. 1i, j; *P* < 0.05), thus resulting in increased mRNA expression of local pro-inflammatory cytokines (e.g., *TNF-α*, *IFN-γ*, *IL-1β*, and *IL-12*) (Fig. 1k, l; *P* < 0.05). Taken together, these data demonstrated that the effect of ZEN-induced toxicity on the reproductive system was linked to an evoked inflammation in the immune system.

### RNA-seq reveals ZEN-induced toxicity on the reproductive system

**Fig. 2:**
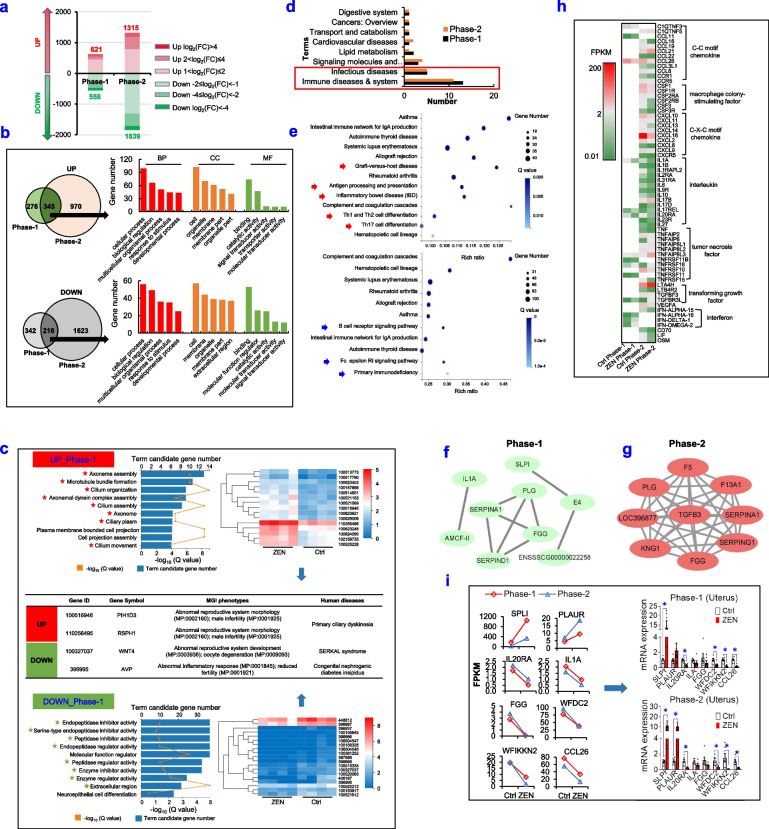
RNA-seq analysis unravels ZEN-induced toxicity on the reproductive system. **a**–**i** ZEN disturbs the reproductive system (e.g., uterus) in pigs during phase 1/2. **a** Distribution of differentially expressed genes (DEGs) in the uterus of phase-1/2’s pig between the Ctrl group and ZEN group (*n*=4). **b** Gene Ontology (GO) classification of overlapping DEGs. BP represents biological process; CC represents cellular component; MF represents molecular function. **c** Top10 terms of GO enrichment of overlapping DEGs in the uterus of phase-1 pig, the heatmap of DEGs enriched in marked terms and MGI & Human diseases of representative DEGs. **d** Statistical analysis of top30 KEGG pathways enriched by DEGs in the uterus. **e** Pathways related to immune diseases and system in top30 KEGG pathways in the uterus of phase-1 (upper) or phase-2 (down) pig. The arrow referred to different terms between phase-1 pigs and phase-2 pigs. **f**, **g** Interactions of genes enriched in immune diseases and system terms in the uterus of phase-1 pigs (**f**) and phase-2 (**g**) pigs. **h** The heatmap of inflammatory cytokines in the uterus of phase-1 and phase-2 pigs based on interactions of genes enriched in immune diseases and system terms. **i** RPKM value and RT-qPCR results of representative genes detected in the uterus. Bar values are means ± SEM. *P < 0.05

Using RNA-seq analysis, we further revealed the underlying mechanism of ZEN-induced toxicity on the reproductive system, including the uterus (Fig. 2, Supplemental Table S3) and ovary (Supplemental Fig. S2, Supplemental Table S3). Compared with the Ctrl group, ZEN exposure significantly shifted uterus’s gene expression patterns, with 1179 and 3154 differential genes in phase-1 pigs and phase-2 pigs, respectively (Fig. 2a). In the uterus from phase-1 pigs that were exposed to ZEN, upregulated genes were 621 while downregulated ones were 558. Additionally, phase-2 pigs that were exposed to ZEN showed 1315 of upregulated genes but 1839 of downregulated ones. Among these, only a few differential genes altered dramatically [log2(FC)>4 or log2(FC)<−4, 6.36~13.98%] (Fig. 2a). Also, there were 345 upregulated and 216 downregulated overlapping genes between phase-1 and phase-2 pigs, and Gene Ontology (GO) analysis of these genes, including BP (biological process), MF (molecular function), and CC (cellular component), showed a similar term enrichment (Fig. 2b). Furthermore, according to the Kyoto Encyclopedia of Genes and Genomes (KEGG) pathway analysis, upregulated overlapping genes were enriched in the cytoskeleton structure, whereas downregulated overlapping genes were involved in protease activity (Fig. 2c). Also, statistical analysis of the top 30 pathways enriched in KEGG demonstrated that the most significant enrichment pathways were related to immune system and infection diseases (Fig. 2d). Among these, both phase-1 pigs (8/13) and phase-2 pigs (8/11) showed many overlaps among most pathways related to immune system, such as autoimmune thyroid disease, primary immunodeficiency, systemic lupus erythematosus, and allograft rejection (Fig. 2d-e).

Next, by using the Cytoscape STRING, we identified that the network of maximal clique centrality (MCC) on coding genes were enriched in immune system and showed three genes (e.g., *serpina1*, *PLG*, and *FGG*) in the first nine genes were consistent between phase-1 pigs and phase-2 pigs (Fig. 2f, g). Although a similar toxicity effect of ZEN exposure on the uterus of pigs during phase 1/2 was shown, its coding genes and underlying mechanism might be different due to age-related changes. As earlier described in Fig. 1, ZEN-induced toxicity caused a swollen vulva and an evoked immune response in the reproductive system, gene expressions of inflammation markers were therefore analyzed in Fig. 2h. Specifically, ZEN exposure significantly lowered the expressions of C-C motif chemokine, macrophage colony-stimulating factor, C-X-C motif chemokine, and interferon, but again dramatically increased those of interleukin, tumor necrosis factor, and transforming growth factor. These results obtained suggested that ZEN exposure aggravated local inflammation in pig’s uterus (Fig. 2h). Accordingly, specific immune-related genes (e.g., *IL-1A*, *IL-20RA*, *CCL26*, *FGG*, *SPLI*, *WFDC2*, *PLAUR*, and *WFIKKN1*) were used for verifying the precision and reproducibility of RNA-seq analysis. As expected, the results from RT-qPCR were basically consistent with RNA-seq data, it, therefore, indicated those with high reliability in its results (Fig. 2i).

In addition to the uterus, a similar effect of ZEN-induced toxicity on immune dysfunction was also observed in the ovary of pigs (Supplemental Fig. S2, Supplemental Table S3). Altogether, the RNA-seq analysis from the reproductive system of pigs during phase 1/2 showed that ZEN-induced toxicity contributed to an evoked inflammation and immune dysfunction.

### RNA-seq analysis unravels ZEN-induced toxicity on the immune system

**Fig. 3:**
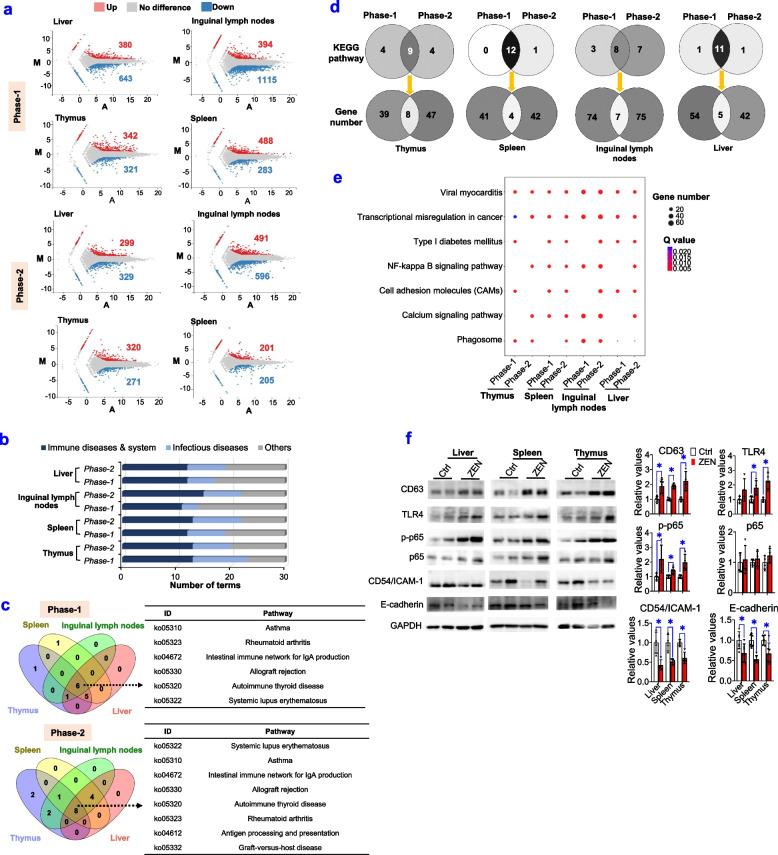
RNA-seq analysis unravels ZEN-induced toxicity on the immune system. **a**–**f** ZEN disturbs the immune system (e.g., liver, spleen, thymus, and inguinal lymph nodes) in pigs, during phase-1/2. **a** Number of DEGs detected in immune organs. **b** Statistical analysis of top30 KEGG pathways enriched by DEGs in immune organs. **c** Venn map of KEGG pathways related to immune disease and system in immune organs. **d** Number of overlapping KEGG pathways between phase-1 and phase-2 pigs and its corresponding number of common genes in overlapping pathways. **e** Other top30 KEGG pathways enriched by DEGs in immune organs. **f** Representative Western blots and its quantifications of key proteins involved in NF-κB (e.g., p-p65), CAMs (e.g., CD54/ICAM-1 and E-cadherin), and phagosome (e.g., CD63 and TLR4) pathways (*n*=4). Bar values are means ± SEM. *P < 0.05

Next, by using RNA-seq analysis, we tested whether ZEN exposure significantly altered gene function and gene expression pattern of the immune system, including liver, spleen, thymus, and inguinal lymph nodes (Fig. 3, Supplemental Table S3). Firstly, a higher number of DEG in the immune system was observed in phase-1 pigs than those in phase-2 pigs (Fig. 3a). According to the top 30 of KEGG pathway enrichment, most pathways were significantly enriched and were mainly involved in immune diseases and system (Fig. 3b). Also, as shown in Fig. 3c, the immune system of pigs during phase 1/2 displayed many overlaps among most pathways, which were related to immune diseases and system. Although both phase-1 and phase-2 pigs that were exposed to ZEN displayed many similar pathways (e.g., immune diseases and systems), there were still great differences in corresponding genes (Fig. 3d). This was consistent with the earlier findings from the reproductive system (Fig. 2).

For both phase-1 and phase-2 pigs, six common pathways were enriched in the immune system, such as asthma, rheumatoid arthritis, the intestinal immune network for IgA production, allograft rejection, autoimmune thyroid disease, and systemic lupus erythematosus (Fig. 3c, Supplemental Table S3). Additionally, two other pathways were also enriched in the immune system of phase-2 pigs, such as antigen processing and presentation, and graft-versus-host disease (Fig. 3c, Supplemental Table S3). Thus, the obtained results indicated that ZEN-induced immune abnormalities had a high similarity and a high tissue specificity for immune organs.

To further analyze the top 30 pathways enriched in KEGG, most pathways were related to immune response and inflammation (Fig. 3e). Among these, immune-related pathways, including phagosome, NF-kappa B, and CAMs, were used to verify the precision and reproducibility of RNA-seq analysis (Fig. 3e, f). Briefly, ZEN-induced toxicity on immune system was as follows: (i) an evoked NF-kappa B pathway (e.g., p-p65); (ii) a stimulated phagosome pathway (e.g., CD63 and TLR4); (iii) a loss of E-cadherin-dependent cell-cell adhesion (e.g., CD54/ICAM-1 and E-cadherin) (Fig. 3f).

Taken together, ZEN exposure, on the one hand, aggregated immune response and inflammation in the immune system by activating the NF-kappa B/phagosome pathways, and on the other hand, resulted in inflammatory lesions by inhibiting cell-cell adhesion.

### ZEN-induced toxicity alters gut microbial metabolites

**Fig. 4:**
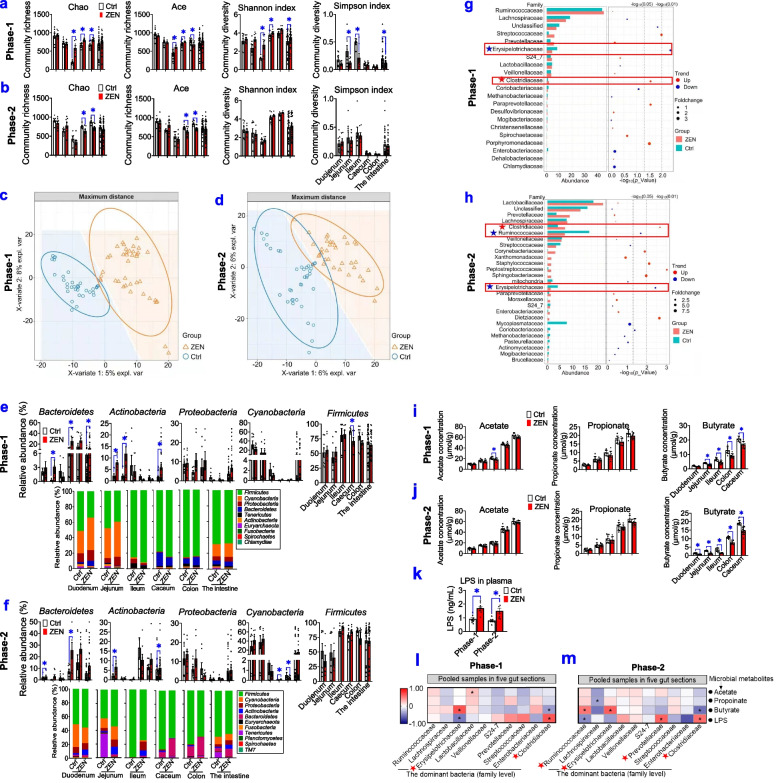
ZEN-induced toxicity alters gut microbial metabolites. **a**, **b** The community richness (Chao and ACE index) and community diversity (Shannon and Simpson index) in all five gut sections, including the duodenum, jejunum, ileum, caecum, and colon of pigs, during phase-1 (**a**) and phase-2 (**b**), between the Ctrl group and ZEN group (*n*=8). **c**, **d** The PLS-DA score plots of phase-1 pigs (**c**) and phase-2 pigs (**d**) between the Ctrl group and ZEN group (*n*=40, pooled samples in five gut sections). **e**, **f** Bacterial compositions at the phylum level and its dominant bacterial phyla (relative abundance > 1.00%) of all five gut sections of phase-1 pigs (**e**) and phase-2 (**f**) pigs that were exposed to ZEN (*n*=8). **g**, **h** Differential abundance analysis of gut bacteria (at the family level) in pigs (*n*=40, pooled samples in five gut sections). The bar plot on the left shows the top 20 most abundant bacteria. The bubble plot on right displays its Wilcox test results. **i**, **j** The concentrations of short-chain fatty acids (SCFAs) in all five gut sections (*n*=8). **k** The levels of plasma lipopolysaccharides (LPS) in pigs (*n*=8). **l**, **m** Spearman’s correlation coefficients between core bacteria (at the family level) and major microbial metabolites, including SCFAs and LPS. Bar values are means ± SEM. *P < 0.05

Given that the gut is a first-line defense against mycotoxin and its gut microbiota represents a crucial bridge between environmental substances and host health, we tested whether ZEN-induced toxicity on the reproductive-immune axis was accompanied by altered gut microbial-derived metabolites (Fig. 4, Supplemental Figs. S3 and S4). Firstly, both phase-1 and phase-2 pigs that exposed to ZEN significantly reduced the richness (e.g., Chao and ACE index) of caecal and colonic microbiota, whereas alterations of α-diversity (e.g., Shannon and Simpson index) were observed in the jejunum and ileum from phase-1 pigs but not phase-2 pigs (Fig. 4a, b). Next, β-diversity analysis was performed to reveal the differences among multiple samples by analyzing the OTUs (97% similarity), and OTU-based PLS-DA analysis showed a clear separation of gut microbiota composition in pigs between the Ctrl group and ZEN group (Fig. 4c, d, Supplemental Fig. S3a-b).

The structural changes of gut microbiota were then evaluated by cluster analysis (Fig. 4e–h, Supplemental Fig. S4). At the phylum level, *Firmicutes* and *Bacteroidetes* were the most abundant phyla in each group (Fig. 4e, f). More specifically, phase-1 pigs that were exposed to ZEN displayed a higher abundance of *Bacteroidetes* in the jejunum, caecum, and intestine, *Actinobacteria* in the duodenum and intestine, and *Firmicutes* in the caecum, respectively (Fig. 4e). Also, significant increases in the relative abundances of *Bacteroidetes* in the duodenum and caecum, *Actinobacteria* in the duodenum and intestine, and *Cyanobacteria* in the caecum and colon were observed in phase-2 pigs that were exposed to ZEN (Fig. 4f). Furthermore, at the family level, compared with the Ctrl group, significant altered gut microbial community (pooled samples in five gut sections) from pigs in the ZEN group were as follows: (1) phase-1 pigs exposed to ZEN showed an increase in *Streptococcaceae* (*P* < 0.05) and *Clostridiaceae* (*P* < 0.05), but a decrease in *Erysipelotrichaceae* (*P* < 0.01) (Fig. 4g); (2) phase-2 pigs exposed to ZEN also displayed an increase in *Clostridiaceae* (*P* < 0.01), whereas a decrease in *Ruminococcaceae* (*P* < 0.05) and *Erysipelotrichaceae* (*P* < 0.01) (Fig. 4h). Collectively, significant changes in α-diversity and β-diversity contributed to an adverse toxicity effect of ZEN on gut bacterial community profiles in both phase-1 and phase-2 pigs (Fig. 4a–h, Supplemental Fig. S4).

In line, we further investigated whether altered gut microbiota affects the production of microbiota-derived metabolites, including short-chain fatty acids (SCFAs) and LPS (an endotoxin derived from the outer membrane of Gram-negative bacteria) (Fig. 4i–k). Intriguingly, although no significant changes were observed in other two SCFAs (acetate and propionate) between the Ctrl group and the ZEN group, reduction of butyrate production was observed in five gut sections from both phase-1 and phase-2 pigs in the ZEN group when compared with those in pigs in the Ctrl group (*P* < 0.05) (Fig. 4i, j). Additionally, ZEN exposure also significantly increased the level of plasma LPS in both phase-1 and phase-2 pigs (Fig. 4k).

Next, Spearman’s correlation test was used for assessing the relationships between the core bacteria (at the family level) and these microbiota-derived metabolites, including SCFAs and LPS (Fig. 4l, m). In phase-1 pigs, the level of butyrate was negatively associated with the relative abundance of *Clostridiaceae* (*ρ* = −0.39, *P* < 0.05) but positively correlated with the relative abundance of *Erysipelotrichaceae* (*ρ* = 0.68, *P* < 0.01) (Fig. 4l). In phase-2 pigs, the concentration of butyrate was negatively linked to the relative abundances of *Lachnospiraceae* (*ρ* = −0.54, *P* < 0.05) and *Clostridiaceae* (*ρ* = −0.56, *P* < 0.05) while positively correlated with the relative abundances of *Ruminococcaceae* (*ρ* = 0.53, *P* < 0.05) and *Erysipelotrichaceae* (*ρ* = 0.63, *P* < 0.01) (Fig. 4m). Also, the concentration of LPS was negatively related to the relative abundances of *Erysipelotrichaceae* (*ρ* = −0.52, *P* < 0.01) in phase-1 pigs, *Ruminococcaceae* (*ρ* = −0.41, *P* < 0.05) and *Prevotellaceae* (*ρ* = −0.45, *P* < 0.05) in phase-2 pigs, but positively linked with the relative abundances of *Clostridiaceae* (*ρ*= 0.66, *P* < 0.05) in both phase-1 and phase-2 pigs (Fig. 4l, m). Accordingly, these data suggested that both *Ruminococcaceae* and *Erysipelotrichaceae* belonged to potential butyrate-producing bacteria, and *Clostridiaceae* might be potential LPS-producing bacteria.

Altogether, these obtained results indicate that ZEN exposure lowered butyrate production by inhibiting butyrate-producing bacteria (e.g., *Ruminococcaceae*, and *Erysipelotrichaceae*), whereas it stimulated LPS production by increasing LPS-producing bacteria (e.g., *Clostridiaceae*).

### ZEN-induced toxicity impairs the intestinal barrier through altered microbial metabolites

**Fig. 5:**
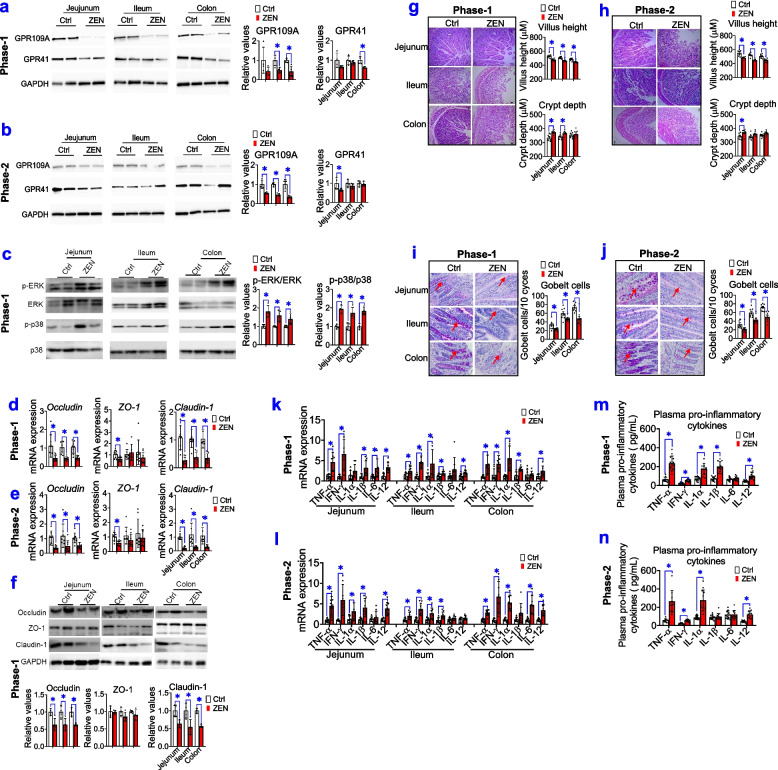
ZEN-induced toxicity impairs the intestinal barrier by altered microbial metabolites. **a**, **b** Representative Western blots and its quantification of SCFAs receptors (e.g., GPR109A and GPR41) in the jejunum, ileum, and colon of pigs, during phase-1 pigs (**a**) and phase-2 pigs (**b**), between the Ctrl group and ZEN group. The GAPDH is used as the loading Ctrl (*n*=4). **c** Representative Western blots and its quantification of the ERK1/2 and p38 signalling pathway (*n*=4). **d**–**f** The mRNA expression (**d, e**, *n*=8**)**, representative Western blots and its quantification (**f***n*=4**)** of tight junction markers (e.g., occludin, claudin-1, and ZO-1) in the jejunum, ileum, and colon. **g**, **h** The intestinal morphology of pigs during phase-1 (**g**) and phase-2 (**h**). The left of panels **g** and **h**: representative light micrographs of a cross-section from the jejunum, ileum, and colon under ×40 magnification. The scale bar represents 200 μm. On the right of panels **g** and **h**: the quantification of villus height and crypt depth (*n*=8). **i**, **j** Periodic acid–Schiff staining (PAS) staining for goblet cells in the jejunum, ileum, and colon. On the left of panels **i** and **j**: representative staining for goblet cells in the intestine (The arrows indicate positive goblet cells). Scale bars: 100 μm. The right of panels **i** and **j**: the quantification of immunoreactive goblet cells using digital image analysis (*n*=8). **k**, **l** The mRNA expression of pro-inflammatory cytokines in the jejunum, ileum, and colon (*n*=8). **m**, **n** The levels of plasma pro-inflammatory cytokines in pigs (*n*=8). Bar values are means ± SEM. *P < 0.05

To identify whether gut microbial metabolites (e.g., butyrate) affect intestinal barrier function, SCFA receptors, signalling pathways in intestinal integrity (e.g., ERK1/2, p38 MAPK, and tight junction markers), intestinal morphology, goblet cells, and pro-inflammatory cytokines were tested (Fig. 5). Firstly, a lower protein expression of GPR109A (*P* < 0.05) but not GPR41 was found in the jejunum, ileum, and colon from both phase-1 and phase-2 pigs that were exposed to ZEN (Fig. 5a, b). Subsequently, decreased butyrate production caused by ZEN exposure significantly evoked the phospho-ERK1/2 and phospho-p38 signalling pathway (Fig. 5c; *P* < 0.05), and then impaired intestinal integrity (e.g., decreased expression of occludin and claudin-1; *P* < 0.05) (Fig. 5d–f) in a GPR109A-dependent manner. Furthermore, impaired intestinal integrity was accompanied by reduction of villus height (Fig. 5g, h; *P* < 0.05) and goblet cells (Fig. 5i, j; *P* < 0.05)]. As a result, ZEN-induced toxicity resulted in intestinal barrier dysfunction, and it then lowered immune defense against ZEN. Indeed, our results also found that ZEN-induced toxicity rapidly increased mRNA expressions of pro-inflammatory cytokines (e.g., *TNF-α*, *IFN-γ*, *IL-1β*, and *IL-12*) (*P* < 0.05) (Fig. 5k, l), thus dramatically aggravating the circulating levels of these pro-inflammatory cytokines (*P <* 0.05) (Fig. 5m, n) and LPS (*P <* 0.05) (Fig. 4k). Accordingly, these data showed that ZEN-induced toxicity impaired the intestinal barrier through altered gut microbial metabolites (e.g., reduction of butyrate production, and increasement of LPS production).

Taken together, we conclude that ZEN-induced toxicity results in impaired intestinal barrier through altered gut microbial metabolites (e.g., decreased butyrate production and/or increased LPS production), and then triggered the systemic inflammatory response, thus disturbing immune defense in the reproductive-immune axis.

### Modified gut microbial metabolites by engineering mycotoxin-degrading enzymes counteract ZEN-induced toxicity on the intestinal barrier

**Fig. 6:**
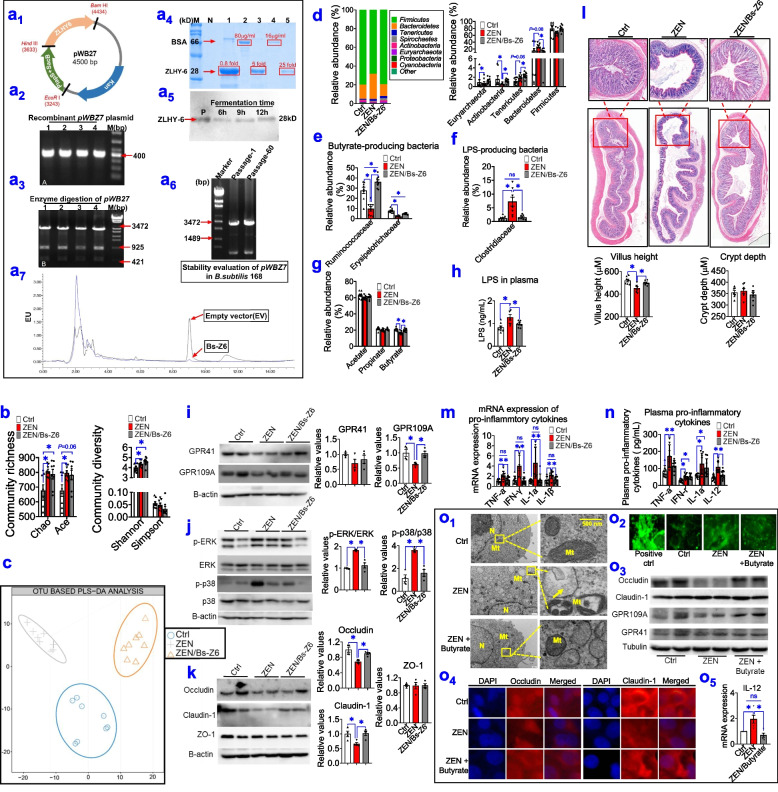
Modified gut microbial metabolites by engineering ZEN-degrading enzyme *ZLHY-6* counteracts ZEN-induced toxicity on intestinal barrier function. **a** Generation of recombinant *B. subtilis*-expressing *ZLHY-6* (the Bs-Z6 strain) and its degradation effect on ZEN-contaminated food. **a**_**1–3**_ Construction and its identification of the recombinant expression vector-*pWBZ7* containing *ZLHY-6* gene (**a**_**1**_). **a**_**2–3**_ The PCR identification and its double enzyme digestion of positive transformants of the recombinant Bs-Z6 strain. Lanes 1-4: positive transformants; M: DNA marker. **a**_**4**_ SDS-PAGE electrophoresis analysis of purified *ZLHY-6* in recombinant Bs-Z6 strain. M: protein ladders; Lanes 1, 3, 5: 0.8, 5, 25-fold target protein purification samples, respectively. Lanes 2, 4: 80μg/mL, 16μg/mL BSA, respectively. **a**_**5**_ SDS-PAGE electrophoresis of recombinant *ZLHY-6* protein expressed in Bs-Z6 at different fermentation duration (6, 9, 12 h). **a**_**6**_ Stability evaluation of *pWBZ7* in the recombinant Bs-Z6 strain. Lanes 1, 2: Enzyme digestion of *pWBZ7* plasmid from the first and 60th generation of this recombinant strain, respectively. M: DNA ladder. **a**_**7**_ The degradation activity of ZEN detected by HPLC for the genetic engineering strains. **b** The community richness (Chao and ACE index) and community diversity (Shannon and Simpson index) in the colon of phase-3 pigs among the control (Ctrl), zearalenone (ZEN), ZEN supplemented with recombinant Bs-Z6 strain (Bs-Z6) groups (*n*=8). **c** Analysis of PLS-DA score plots (*n*=8). **d** Bacterial compositions at the phylum level and its dominant bacterial phyla (relative abundance > 1%). **e**, **f** Differential abundance analysis of potential butyrate-producing bacteria and LPS-producing bacteria (at the family level) (*n*=8). **g** The concentrations of SCFAs in the colon. **h** The levels of plasma LPS. **i**–**k** Representative Western blots and its quantification of SCFAs receptors (GPR109A and GPR41), the ERK1/2 and p38 signalling pathway, and tight junction markers (occludin, claudin-1, and ZO-1) in the colon (*n*=4). The B-actin is used as the loading control (*n*=4). **l** Representative light micrographs of a cross-section of the colon under ×40 magnification and its quantification of villus height, crypt depth (*n*=8). Scale bar represents 200 μm. **m**, **n** The mRNA expression (**m**) and plasma concentrations (**n**) of pro-inflammatory cytokines (*n*=8). **o** Butyrate supplementation reduces ZEN-induced toxicity on intestinal barrier in porcine intestinal epithelial cell (IPEC-J2). **o**_**1**_ Representative TEM images of IPEC-J2 cells from each group (The arrows show swelling nucleus and/or mitochondria). The scale bar represents 500 nm. **o**_**2**_ Representative confocal microscope images of oxidative stress marker ROS content) of IPEC-J2 cells. **o**_**3–4**_ Representative Western blots and confocal microscope images of tight junction markers (occludin and claudin-1) (*n*=3). **o**_**5**_ The mRNA expression of pro-inflammatory cytokines (e.g.,* IL-12*) (*n*=4). Bar values are means ± SEM. *P < 0.05

To validate these findings above, we generated recombinant *Bacillus subtilis* (*B. subtilis*) 168-expressing ZEN-degrading enzyme *ZLHY-6* [28] as an example to investigate the feasibility of enzymatic removal of ZEN from mycotoxin-contaminated food. Briefly, we constructed recombinant expression vector *pWBZ7* (Fig. 6a_1_–a_3_), which regulated ZEN degradation enzyme gene *ZLHY-6* by the promoter *P*_*lapS*_ (Supplemental Fig. S5a-c). And, the *pWBZ7* plasmid was then electrically transformed into *B. subtilis* 168, which was designated as Bs-Z6 in this study (Supplemental Fig. S5b). Using BSA as a protein standard, we estimated the concentration of recombinant *ZLHY-6* protein expressed in Bs-Z6 and found it could reach the peak concentration of 500 μg/mL at a fermentation time of 12 h (Fig. 6a_4_–a_5_). To further evaluate the stability of *pWBZ7* in recombinant Bs-Z6 strain, the plasmids on the first passage and the 60th passage were digested with two enzymes: *EcoR I* and *BamH I*. The results showed that the plasmids of the two passages were cut into fragments with sizes of 3310 and 1190 bp, respectively, indicating that recombinant *pWBZ7* plasmid was highly stable in recombinant Bs-Z6 strain (Fig. 6a_6_). Of note, after 12 h of incubation at 37° C, the degradation effects of recombinant Bs-Z6 strain on ZEN-contaminated food (e.g., corn) reached above 92% (Fig. 6a_7_, Supplemental Fig. S5d-e). Also, the relative enzyme activity recorded was 219.02 U mL^−1^ at the end of 12 h (Supplemental Fig. S5f). Consistent with these data, after optimizing fermentation conditions (e.g., dissolved O_2_, pH, and temperature) (Supplemental Fig. S5g), the highest protein expression of *ZLHY6* in recombinant Bs-Z6 strain was also recorded at 12 h (Supplemental Fig. S5h).

Subsequently, using the pig (phase 3) as an animal model, we identified whether a ZEN-contaminated diet supplemented with recombinant Bs-Z6 strain could counteract ZEN-induced toxicity on the intestinal barrier by regulating colonic microbial metabolites (Fig. 6b–n, Supplemental Fig. S6). When a ZEN-contaminated diet was combined with recombinant Bs-Z6 strain, by using the HPLC-MS/MS assay, we found that ZEN/its metabolites residues were significantly decreased in intestinal contents, blood, liver, and uterus (*P* < 0.05) (Supplemental Table S4). Thus, these findings further supported the degradation effects of recombinant Bs-Z6 strain on ZEN-contaminated food.

As earlier observed (Fig. 4a, b), ZEN exposure resulted in significant alterations in the richness (e.g., Chao and ACE index) and α-diversity (e.g., Shannon index) (*P* < 0.05) (Fig. 6b). However, no statistical differences were found in these parameters between the ZEN group and the ZEN/Bs-Z6 group (Fig. 6b). Using OTU-based PLS-DA analysis, we showed ZEN exposure resulted in an obvious change of β-diversity, which was prevented by recombinant Bs-Z6 strain (Fig. 6c). Furthermore, by using cluster analysis, we investigated the toxicity effects of recombinant Bs-Z6 strain on structural changes of gut microbiota (Fig. 6d, Supplemental Fig. S6). As shown in Fig. 6d, ZEN exposure significantly changed the three most dominant phyla (*Actinobacteria*, *Tenericutes*, and *Bacteroidetes*), which was also counteracted by this recombinant strain (*P* < 0.05). In agreement with earlier descriptions (Fig. 4g, h, l, m), ZEN exposure significantly lowered potential butyrate-producing bacteria (e.g., *Ruminococcaceae* and *Erysipelotrichaceae*) (*P* < 0.05), while increased potential LPS-producing bacteria (e.g., *Clostridiaceae*) (*P* < 0.05). Notably, these altered dominant bacteria were preserved by recombinant Bs-Z6 strain (Fig. 6e, f).

Considering that ZEN exposure significant altered gut microbial metabolites, we also observed that recombinant Bs-Z6 strain counteracted ZEN-induced a loss of butyrate (Fig. 6g; *P* < 0.05), whereas inhibited ZEN-induced an increase of LPS (Fig. 6h; *P* < 0.05). As previously described (Fig. 5), reduction of butyrate production resulted in a lower protein expression of SCFA receptors (e.g., GPR109A), and an increase in phosphorylation of ERK1/2 and p38, thus leading to impaired intestinal integrity (e.g., decreased expression of occludin and claudin-1) as well as decreased villus height. These abnormalities in the intestinal barrier were all restored upon increased butyrate production, which was achieved by recombinant Bs-Z6 strain (Fig. 6i–l; *P* < 0.05). Furthermore, this restored intestinal barrier function significantly lowered the mRNA expression and the circulating level of pro-inflammatory cytokines (e.g., TNF-α, IFN-γ, IL-1α, and IL-1β), thus leading to increased immune defense against ZEN (Fig. 6m, n; *P* < 0.05).

To further confirm these findings above, the porcine small intestinal epithelial cell line, IPEC-J2, is also employed in this study (Fig. 6o). Using the TEM method, we observed that ZEN exposure resulted in mitochondrial dysfunction and increased apoptosis in cells, which was rescued by butyrate supplementation (Fig. 6o_1_). Additionally, in IPEC-J2 cells, ZEN exposure increased ROS content (Fig. 6o_2_) and pro-inflammatory cytokines (e.g., IL-12; *P* < 0.05) (Fig. 6o_5_), but decreased the expression of tight junction markers (e.g., occludin; *P* < 0.05) (Fig. 6o_3-4_). As expected, when ZEN exposure was combined with butyrate, these ZEN-induced abnormalities were also partially preserved (Fig. 6o_1-5_).

Taken together, both *in vitro* and *in vivo* data demonstrate that modified gut microbial metabolites (e.g., increased butyrate production and/ or decreased LPS level) offer the potential strategy to counteract ZEN-induced toxicity on the intestinal barrier and its immune dysfunction.

### Modified gut microbial metabolites reduces ZEN-induced toxicity on reproductive and immune systems

Next, we investigated whether gut modified microbial metabolites, which was achieved by the supplementation of recombinant Bs-Z6 strain, counteracted ZEN-induced toxicity on the reproductive-immune axis in pigs during phase-3.

**Fig. 7:**
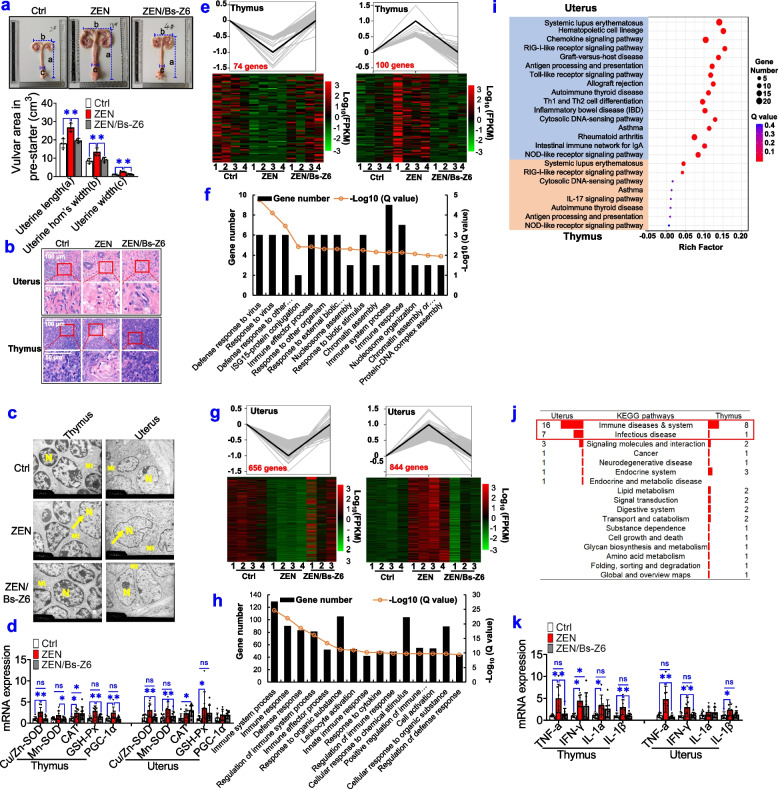
Modified microbial metabolites reduce ZEN-induced toxicity on reproductive and immune systems. **a** Uterine size (uterine length, uterine width, and uterine horn’s width) of phase-3 pigs among the control (Ctrl), zearalenone (ZEN), ZEN supplemented with recombinant Bs-Z6 strain (Bs-Z6) groups (*n*=4). **b** Representative H&E staining of immune organs (e.g., thymus) and reproductive organs (e.g., uterus) from each group under ×40 magnification. The scale bar represents 100 μm. **c** Representative TEM images of thymus and uterus (The arrows show swelling nucleus). The scale bar represents 2 μm. **d** The mRNA expression of oxidative stress markers in the thymus and uterus (*n*=8). **e**, **g** Transcriptomic analysis reveals altered gene expression patterns in the thymus (**e**) and uterus (**g**). **f**, **h** The top 15 enriched Gene Ontology (GO) terms from the biological process in the thymus (**f**) and uterus (**h**). **i** Pathways related to immune diseases and system in top30 KEGG pathways of the thymus and uterus. **j** Statistical analysis of top30 KEGG pathways enriched by differentially expressed genes (DEGs). **k** RT-qPCR results of pro-inflammatory cytokines detected in the thymus and uterus. Bar values are means ± SEM. *P < 0.05

Firstly, no significant changes were observed in pig's growth performance among all groups (Supplemental Table S5). As indicated in Fig. 7a and Supplemental Fig. S7, ZEN exposure resulted in a larger vulvar area and uterine size (uterine length, uterine width, and uterine horn’s width), which was completely restored by recombinant Bs-Z6 strain (*P* < 0.05). Using the HE staining and TEM methods, we also observed that ZEN exposure contributed to obvious inflammatory lesions, swelling nucleus, mitochondrial oxidative stress in immune organ (e.g., thymus) and reproductive organ (e.g., uterus), which were preserved by recombinant Bs-Z6 strain (Fig. 7b,c). Also, in agreement with earlier findings in Fig. 1k, l, mRNA expression of oxidative stress markers (e.g., *Cu/Zn-SOD*, *Mn-SOD*, *GSH-Px*, and *PGC-1α*) was significantly increased upon ZEN exposure, while was completely rescued when ZEN exposure was combined with recombinant Bs-Z6 strain (Fig. 7d).

Also, by using RNA-seq analysis, we examined whether ZEN-contaminated food supplemented with recombinant Bs-Z6 strain counteracted ZEN-induced toxicity on the reproductive-immune axis in pigs during phase-3 (Fig. 7e–j, Supplemental Fig. 8). For altered gene expression pattern, we identified that 74 downregulated genes and 100 upregulated genes in immune organ (e.g., thymus) could be preserved by recombinant Bs-Z6 strain (Fig. 7e). Also, 656 downregulated genes and 844 upregulated genes in reproductive organ (e.g., uterus) were rescued by this recombinant strain (Fig. 7g). According to the top 15 enriched GO terms from biological process, most ZEN-induced downregulated genes that were rescued by recombinant Bs-Z6 strain, were closely related to an immune system process, immune response, and defense response (Fig. 7f, h). To further analyze the top 30 pathways enriched in KEGG, most pathways are also related to immune systems and infection disease (9/26 in the thymus; 23/30 in the uterus) (Fig. 7i, j). Among these, immune-related pathways were commonly enriched in both the thymus and uterus, such as, systemic lupus erythematosus, RIG-I-like receptor signalling pathway, autoimmune thyroid disease, antigen processing and presentation, and NOD-like receptor signalling pathway (Fig. 7i, j). In agreement with earlier findings (Fig. 1k, l), mRNA expression of pro-inflammatory cytokines (e.g., *TNF-α*, *IFN-γ*, *IL-1α*, and *IL-1β*) was significantly increased upon ZEN exposure, but was completely inhibited when ZEN exposure was combined with recombinant Bs-Z6 strain (Fig. 7k).

Taken together, these data obtained further supported that ZEN-induced intestinal barrier dysfunction resulted in the systemic inflammation through altered microbial metabolites, thus disturbing immune defense in the reproductive-immune axis. Notably, the regulation of gut microbial metabolites (e.g., butyrate and LPS), which was achieved by recombinant Bs-Z6 strain, could counteract ZEN-induced toxicity on the reproductive-immune axis.

## Discussion

**Fig. 8:**
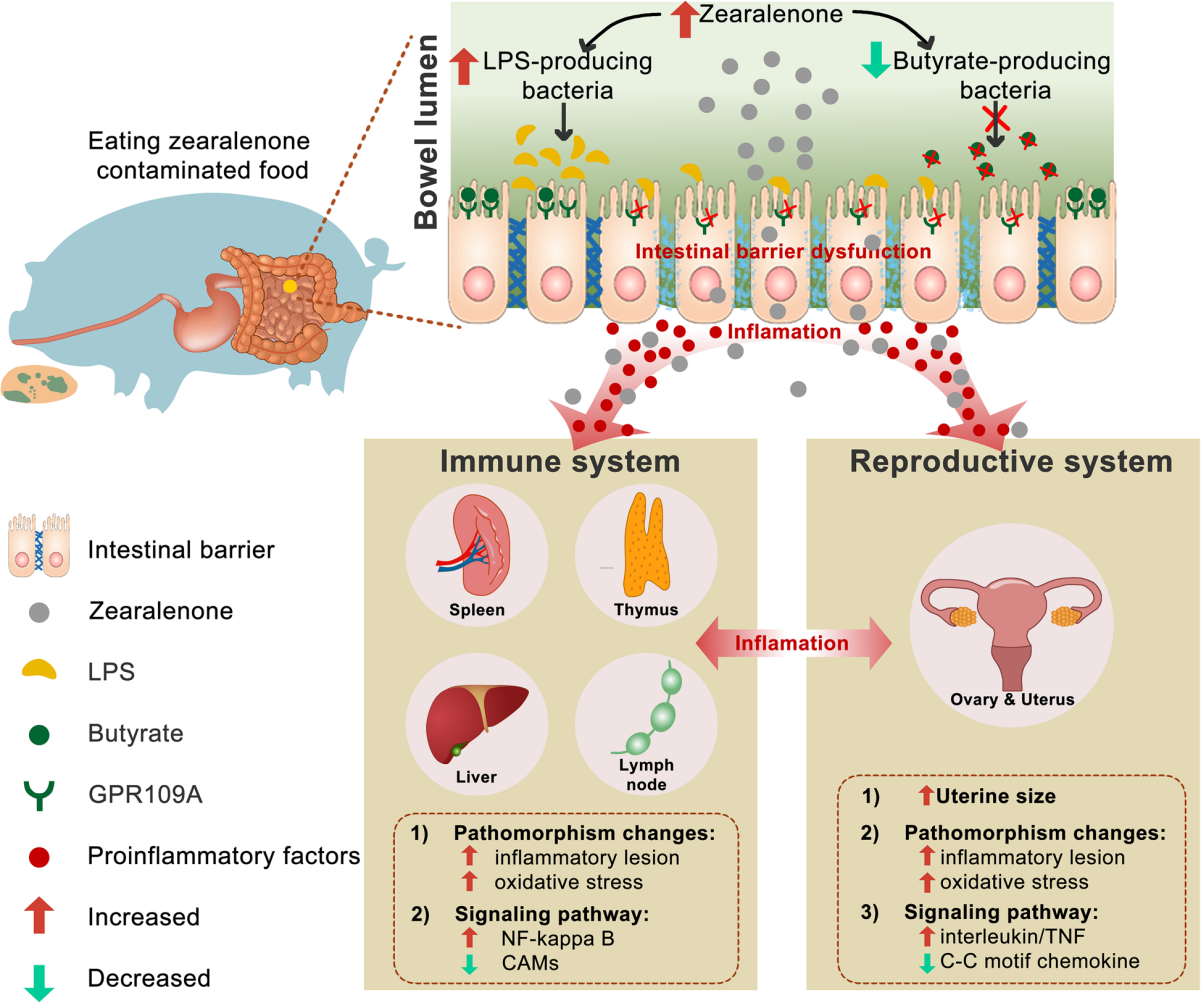
Schematic representation of ZEN disturbs the reproductive-immune axis accompanied by altered gut microbial metabolites. (1) Excessive mycotoxin (e.g., ZEN)-induced toxicity firstly shifted gut bacterial community profiles, and then elevated microbial-derived LPS but lowered microbial-derived butyrate. (2) Reduction of butyrate production resulted in intestinal barrier dysfunction via a GPR109A-dependent manner, and together with increased LPS in plasma aggravated the systemic inflammation. (3) Subsequently, ZEN-induced systemic inflammation impaired immune-related pathways in immune systems, and these changes were also accompanied by pathomorphism and mitochondrial oxidative stress. (4) Meanwhile, ZEN-induced toxicity disturbed the reproductive system through an over-triggered systemic inflammatory response, which was closely related to alterations in specific immune-related genes

Here, by using the pig (during the three phases) as an important animal model, the present results obtained led us to formulate the following observations (see Fig. 8): (i) ZEN-induced toxicity firstly disturbed gut bacterial community profiles, and lowered butyrate production by inhibiting butyrate-producing bacteria (e.g., *Ruminococcaceae*, and *Erysipelotrichaceae*), while increased LPS production by enhancing LPS-producing bacteria (e.g., *Clostridiaceae*). ii) Reduction of butyrate production resulted in intestinal barrier dysfunction via a GPR109A-dependent manner, and together with increasement of LPS production in plasma then aggravated the systemic inflammation. iii) Subsequently, ZEN-induced systemic inflammation impaired immune-related pathways in immune systems, and these changes were also accompanied by inflammatory lesions and mitochondrial oxidative stress; iv) Meanwhile, ZEN-induced toxicity harmed the reproductive system through an over-triggered systemic inflammatory response, which was closely related to alterations in specific immune-related genes. Altogether, we concluded that ZEN exposure impaired intestinal barrier function, and then triggered the systemic inflammatory response via altered gut microbiota-derived metabolites (e.g., decreased butyrate production, and increased LPS production), thus directly and/or indirectly disturbing immune defense in the reproductive-immune axis. Accordingly, three possible explanations are highlighted as follows:

(i) As revealed by increasement of pro-inflammatory cytokines in plasma (Figs. 5m, n and 6n), it indicates that ZEN exposure contributes to the systemic inflammatory response. Briefly, ZEN exposure lowers gut microbiota-derived butyrate, and then impairs intestinal barrier via a GPR109A-dependent manner, thus aggravating the local intestinal inflammation. This results in acute increase in levels of pro-inflammatory circulating cytokines and then facilitates the onset of the injury and inflammation in both immune and reproductive systems. (ii) Also, as evidenced by the moderately increased LPS concentration in plasma (Figs. 4k and 6h), ZEN-induced intestinal leakage may allow for other microbial products such as LPS to transfer into immune and reproductive organs, thus resulting in an impaired immune defense [29, 30]. (iii) Indeed, as observed by increased residue of ZEN/its metabolites in five gut sections, blood, immune, and reproductive organs (Supplemental Tables S1 and S4), it suggests that ZEN and its metabolites can directly disturb immune defense in the reproductive-immune axis. Briefly, ZEN is firstly absorbed and metabolized in the intestine (a frequent route of exposure to this toxin), in which residues of ZEN and its metabolites are directly transferred into reproductive and immune organs via the systemic circulation, thereby leading to that ZEN and/or its metabolites itself has toxicity effects on immune and reproductive systems via its biotransformation reactions.

For the first two explanations, by using in vivo validation experiment, we showed that modified microbial metabolites (e.g., increased microbiota-derived butyrate, decreased microbiota-derived LPS), which was achieved by recombinant *B. subtilis*-expressing the ZEN-degrading enzyme (*ZLHY-6*), could counteract ZEN-induced toxicity on intestinal barrier function. Of note, this restored intestinal barrier function was also independent of a GPR109A manner. Also, using a cell model (e.g., IPEC-J2), we observed butyrate supplementation partially rescued ZEN-induced abnormalities in the intestinal barrier function. Subsequently, restored intestinal barrier function, which was regulated by modified microbial metabolites, blunted local intestinal inflammation, and then boosted the systemic immune defense against ZEN-induced toxicity in the reproductive-immune axis. In agreement with this scenario, in addition to important roles in maintaining intestinal homeostasis, gut microbial metabolites also affect multitudinous physiological functions of other visceral organs and are involved in mediating the pathogenesis of different diseases in these organs [29, 31–34]. Intestinal dysbiosis may result in intestinal inflammation, along with chronic inflammation in organs outside the intestine (e.g., liver and kidney) [35]. Also, recent advances in research of understanding a relationship referred to the gut−liver axis found gut microbiota-derived metabolites (e.g., SCFAs) were closely connected to the liver through the portal circulation [36]. In the case of an impaired intestinal barrier, microbiota-derived LPS together with the resultant molecules can transfer into the liver, leading to liver injury and inflammation [29, 30]. Together with our findings, we provide a basic mechanistic explanation of ZEN-induced intestinal barrier dysfunction through microbiota-derived metabolites that underlies the onset of the systemic inflammation and disturb immune and reproductive organs.

With respect to the third explanation, following ingestion and absorption in the intestine, biotransformation reactions of ZEN or other *Fusarium* mycotoxins are presented and metabolized mainly by the bile, urine, plasma, liver, and uterus, which are probably transferred by intestinal mucosa or erythrocytes [5, 37]. Using the HPLC-MS/MS assay, ZEN and its metabolites were detected mainly in the intestine, and then transferred into other visceral organs (e.g., immune and reproductive organs) via the blood circulatory system. Considering ZEN-induced toxicity on the reproductive system, one endocrine-disrupting effect of ZEN and/or its metabolites might be associated with the disruption of hormone production by the pituitary gland [5, 17]. Prepubertal exposure to dietary ZEN might cause premature activation of the hypothalamic kisspeptin-GPR54 signalling pathway, which leads to the advancement of vaginal opening and enlargement of the uterus at the rat’s periphery [38]. A second effect, and the best documented, is associated with gonad function. ZEN is reported to modulate the activity of ovaries in animal models [5]. In female mice, the effect of ZEN on the endocrine system is closely associated with disturbances in oocyte production and maturation through cell cycle arrest [39], DNA methylation [40], cytoskeleton modulation, and proliferation of granulosa cells [40]. In this study, it is conceivable that ZEN-induced toxicity dramatically results in swelling vulvar area and augmented mitochondrial oxidative stress, thus impairing immune defense in pigs during the three phases.

Furthermore, ZEN-induced toxicity has also been responsible for the pathogenesis of the atrophy, and cell depletion in the immune system, as resulted from the present finding. This is consistent with previous studies related to ZEN exposure. In SD pregnant rats, ZEN treatment for 7 days lowered the viability of splenocytes and T-cell proliferation and induced histopathological damage in the spleen, this is accompanied by increased IL-6, IL-18, and IL-1β [41]. In BALB/c mice (female, 7-week-old), ZEN treatment for 2 weeks also increased CD19^+^ and CD11c^+^ in the mesenteric lymph nodes, and TNF-α and apoptosis in the spleen, but again with decreased CD4^+^, CD8^+^, and F4/80^+^ in both mesenteric lymph nodes and spleen [42]. In pigs, 1.1, 2.0, and 3.2 mg/kg BW of ZEN treatment for 18 days resulted in increased IL-1β and IL-6, cytoplasmic edema, and splenic damages [43]. In agreement with this, RNA-seq analysis from the immune system further demonstrated that ZEN-induced toxicity not only impaired immune defense by evoking NF-kappa B and phagosome pathways, but also resulted in inflammatory lesions by inhibiting cell-cell adhesion. Collectively, ZEN itself had toxicity effects on immune defense in the reproductive-immune axis, this might be due to that the residues of ZEN and/or its metabolites in the intestine are directly transferred into these extraintestinal organs. However, using isotope-coded ZEN-labeling strategies to track its underlying mechanism needs to be studied further.

## Conclusions

Together with our findings in pigs during three growth phases, we highlighted a potential mechanism linking gut microbiota-derived metabolites (e.g., butyrate, LPS) to ZEN-induced impaired immune defense in the gut-reproductive-immune axis through the systemic inflammation. Of note, the regulation of gut microbial metabolites (e.g., increased butyrate production and/or decreased LPS production) through *B. subtilis* 168-expressing ZEN-degrading enzyme *ZLHY-6* could counteract ZEN-induced toxicity on the gut-reproductive-immune axis. Herein, this comprehensive study sheds light on a novel toxicity mechanism of ZEN in the gut-reproductive-immune axis, and it suggests that targeting gut microbial metabolites is a promising strategy to overcome potential threats to farm animals and humans through the food chain.

## Materials and methods

Materials/reagents in this study are listed with catalog numbers and vendors in Supplemental Table S6.

### Generation of recombinant *B. subtilis*-expressing *ZLHY-6*

ZEN-degrading gene *ZLHY-6* was cloned from C. rosea 31535 by our laboratory [28] and submitted to GenBank (Accession No. HQ825318.1). To enhance the secretory expression, both MFα signal peptide (*SacB*) and promoter *P*_*lapS*_ were inserted into the recombinant *pWBZ1 *vector (it contains ZEN-degrading gene *ZLHY-6*), which was previously constructed in our laboratory [28]. In this study, the recombinant plasmid was designated as the *pWBZ7* plasmid. Briefly, the codon-optimized version of MFα signal peptide (*SacB*) and promoter *P*_*lapS*_ were synthesized by BGI (Huada Gene Institute) Genomics (Shenzhen, China) based on *B. subtilis* codon preference for reaching a high protein expression.

Reverse transcription and PCR were carried out with the forward prime 5′-CCGGAATTCTCAGGAGCATTTAACCTAAA-3′ and the reverse primer 5′- GGCAAAAGCTTGAGTTGC -3′ to amplify the complementary DNA (cDNA) sequence of the codon-optimized version of MFα signal peptide (*SacB*) and promoter *P*_*lapS*_. The applied PCR conditions were as follows: 94 °C (5 min), followed by 31 cycles of 94 °C (1 min), 50 °C (30 s), and 68 °C (30 s), with a final extension at 68 °C (5 min). And then, the primers amplified the cDNA product, which was verified by 2% agarose gel electrophoresis. The resulting PCR product was gel-purified using the E.Z.N.A.TM Gel Extraction Kit (Omega Co., USA) and ligated into the* pWBZ1* vector to generate a new recombinant *pWBZ7* plasmid through two enzymes: *ECOR I* and *BamH I*. The aforementioned recombinant plasmid of *pWBZ7* contains ZEN-degrading gene *ZLHY-6*, MFα signal peptide (*SacB*), promoter *P*_*lapS*_, a versatile multiple-cloning site, and a kanamycin-resistant gene for selection. Finally, the recombinant *pWBZ7* plasmid were electrically (2.2 kV, 25μF, 200Ω) transformed into *B. subtilis* 168, which was designated as Bs-Z6 in this study. Additionally, the original vector, without the ZEN-degrading gene *ZLHY*-6 insert, was also transformed into *B. subtilis* 168 as a control, which was designated empty vector (EV).

### Growth and fermentation of the recombinant Bs-Z6 strain

The recombinant Bs-Z6 strain was streaked on 5 mL LB (Luria-Bertani) liquid medium containing kanamycin (24 h, 37 °C, 220 rpm). The OD_600_ of the overnight culture was then determined to evaluate the growth of the recombinant Bs-Z6 strain. To determine its enzyme activity, the recombinant Bs-Z6 strain was cultured at 37 °C and 220 rpm for 2, 4, 6, 8, 10, 12, 15, 23, 30, 35, 47, and 54 h, respectively. Also, by using different concentrations of BSA as a protein standard, we then estimated the concentration of recombinant *ZLHY-6* expressed in recombinant Bs-Z6 strain (Fig. 6a_4_–a_5_). Also, we evaluated the stability of *pWBZ7* in this recombinant strain from the first passage and the 60th passage, which were digested with two enzymes*: ECOR I* and *BamH I*. Moreover, the degradation effects of recombinant Bs-Z6 strain on ZEN-contaminated food (e.g., corn) were also evaluated after 12 h of incubation at 37°C.

Finally, to identify optimized fermentation conditions (e.g., dissolved O_2_, pH, and temperature), the recombinant Bs-Z6 strain was fermented in a 20L-continuous stirred-tank reactor under different fermentation times (0–36 h).

### Animal experiments

All the procedures in this study were approved by the Animal Welfare Committee of the Institute of Subtropical Agriculture, Chinese Academy of Sciences and under protocol no. 2016/06/15/013. As suggested by the animal welfare protocol, all efforts were made to minimize animal suffering and to use only the number of animals necessary to produce reliable scientific data.

### Experiment design

According to China Feed Hygiene Standard (GB 13078-2017), ZEN maximum limit to pigs is less than 150 μg/kg. In this study, the corn husk hydrolysate without and/or with ZEN that provided by Neimenggu Fufeng Biotechnologies Co., Ltd (Neimenggu, China) was employed to form the diet without and/or with ZEN, respectively. The current experiment designs were as follows:

(i) During phase-1, a total of 48 raised Landrace-Duroc pigs (body weight: 8.87 ± 0.43 kg) obtained from the Shenzhen PremixINVE Nutrition Co., LTD (Shenzhen, China) were randomly assigned to the following two groups that were provided: (a) the basal diet containing 2% corn husk hydrolysate (the concentration of ZEN ≤150 μg in per kg diet; the Ctrl group), (b) the basal diet containing 2% corn husk hydrolysate (the concentration of ZEN is 1687.42 ± 58.93 μg in per kg diet; the ZEN group).
(ii) During phase-2, a total of 48 pigs (body weight: 19.97 ± 0.73 kg) were also randomly assigned to the following two groups that were provided: (1) the basal diet containing 5% corn husk hydrolysate (the concentration of ZEN ≤150 μg in per kg diet; the Ctrl group), (2) the basal diet containing 5% corn husk hydrolysate (the concentration of ZEN is 3684.83 ± 40.52 μg in per kg diet; the ZEN group).
(iii) To confirm the results above, during phase 3, a total of 36 pigs (body weight: 63.77 ± 3.97 kg) were randomly assigned to the following three groups that were provided: (1) the basal diet containing 8% corn husk hydrolysate (the concentration of ZEN ≤150 μg in per kg diet; the Ctrl group), (2) the basal diet containing 8% corn husk hydrolysate (the concentration of ZEN is 8023.81 ± 231.66 μg in per kg diet; the ZEN group), (3) the ZEN group supplemented with recombinant *B. subtilis*-expressing ZLHY-6 (the concentration of ZEN is less than 250 μg in per kg diet; the ZEN/Bs-Z6 group). As previously described, the concentration of ZEN-degrading protein-expressed *B. subtilis* was 500 μg /L. Throughout the experiment trial, the diets per kg of the ZEN/Bs-Z6 groups were supplemented with 20 mg/kg lyophilized powder of recombinant *B. subtilis*-expressing ZLHY-6 (Bs-Z6), and both the Ctrl and ZEN groups were only given the empty vector-expressing *B. subtilis* (the same volume).

### Animal management and sample collection

The pigs during the three phases had ad libitum access to the water and feed, and then remaining feed was weighed at 0800 h per day. Average daily feed intake and body weigh were recorded weekly to assess the feed-to-gain ratio and average daily gain. In addition, the signs of diarrhea, sickness, and abnormal behavior were also recorded throughout the experimental period [44].

At the end of the experiment, two pigs from each pen were chosen at random, a 10-mL blood sample was collected from the anterior vena cava, and were then sacrificed with sodium pentobarbital (50 mg/kg BW) [45]. The plasma was collected by centrifugation at 3000*g* for 15 min at 4 °C, and then stored at −20 °C until further analysis. Immediately after slaughter, the following tissue samples were collected from eight pigs of each group and were chosen at random: five gut-associated sections (duodenum, jejunum, ileum, caecum, and colon), immune organs (e.g., liver, spleen, thymus, and inguinal lymph nodes), and reproductive organs (e.g., uterus and ovaries). The samples were snap-frozen in liquid nitrogen. For both hematoxylin and eosin (HE) staining and periodic acid–Schiff (PAS) staining, the samples were fixed in % paraformaldehyde (PFA) solution. Also, for transmission electron microscope (TEM), the samples were kept in 4% formaldehyde solution. The rest of the samples were snap-frozen in liquid nitrogen and were kept frozen until further use.

### Sequence processing and analysis of gut microbiota

Sequence processing and analysis of gut microbiota were prepared according to our previous description [46]. Briefly, genomic DNA was extracted from digesta samples in five gut-associated sections (duodenum, jejunum, ileum, caecum, and colon) the E.Z.N.A.® Stool DNA Kit (Omega Bio-tek, Inc., Norcross, GA, USA) in accordance with the manufacturer’s instructions. Quality of total DNA was verified using electrophoresis analysis.

The PCR system was configured using 30 ng genomic DNA samples of known quality and the corresponding fusion primers to set the PCR parameters for amplification. The V_3_–V_4_ region of 16S rRNA gene was then amplified with primers (F: 5′-ACTCCTACGGGAGGCAGCAG-3′ and R: 5′-GGACTACHVGGGTWTCTAAT-3′) by PCR. The forward and reverse primers were tagged with Illumina adapter, pad, and linker sequences. The PCR enrichment was performed in a 50-μL reaction volume containing 30 ng of template, fusion PCR primers, and PCR master mix. The cycling conditions were as follows: 95 °C for 3 min, 30 cycles of 95 °C for 45 s, 56 °C for 45 s, and 72 °C for 45 s, and a final extension at 72 °C for 10 min. The PCR products were purified using Agencourt AMPure XP (Indianapolis, Indiana, USA) beads and eluted using an elution buffer (Omega Bio-tek, Norcross). Libraries were validated using an Agilent Technologies 2100 bioanalyzer (Agilent, Santa Clara, CA, USA) before being sequenced on an Illumina HiSeq 2500 platform (Illumina, San Diego, CA, USA) according to standard Illumina pipelines to generate 2 × 250 bp paired-end reads. Data were filtered by removing low-quality reads in each 25-bp window. Briefly, the entire sequence was removed if the final base was truncated from the window with an average quality of < 20 and if the read length after truncation was 75% lower than the original read length. Joint contamination reads, N-containing reads, and low complexity reads were also removed to obtain high-quality clean data. All bacterial 16S rRNA gene amplification, cloning, and sequencing of the PCR products were performed at BGI (Huada Gene Institute) Genomics (Shenzhen, China). Paired-end reads from the original DNA fragments were merged by FLASH. The resulting labels were assigned to the operational taxonomic units (OTUs) with a threshold value of 97%. The complexity of species diversity (α-diversity) was analyzed by calculating Chao, Shannon, ACE, and Simpson indices using Mothur (version v.1.30.13). Variation in the community composition was measured by calculating partial least squares-discriminant analysis (PLS-DA). Additionally, the relative abundance of dominant bacteria at the phylum, family, and genus levels was also analyzed. Also, differential abundance analysis and Wilcox test of gut bacteria (at the family level) were conducted.

### Analysis of SCFA concentrations

The analysis of SCFAs concentrations was prepared according to our previous description [47]. Briefly, approximately 300 mg digesta samples in five gut-associated sections (duodenum, jejunum, ileum, caecum, and colon) were acidified to pH 2.2 using 25% metaphosphoric acid solution and centrifuged at 4800×*g* for 20 min at 4°C and the internal standard (100 mM quinic acid) was added. After mixing by vortex for 30 s and standing at 20°C for 10 min, the centrifugation was repeated (4800*g*, 4°C for 20 min), and the supernatants were then analyzed for SCFAs with an Agilent 6890 gas chromatograph (Agilent Technologies, Inc., Palo Alto, CA). All samples and SCFA standards (e.g., acetate, propionate, and butyrate) were separated on an HPLC-C_18_ column. The analytical method followed a previous publication [47]. Each sample was detected in triplicate, and calibration curves based on the concentrations and peak areas of SCFA standards were used to assess the concentrations of individual SCFAs.

### High-throughput RNA sequencing and bioinformatic analysis

RNA-seq and bioinformatics analyses were performed based on the processes used in our previous description [48]. Total RNAs were extracted from the immune system (e.g., liver, spleen, thymus, and inguinal lymph nodes) and reproductive organs (e.g., uterus and ovaries) of pigs, during phase-1 and phase-2, between the Ctrl group and ZEN group with TRIzol Reagent (Invitrogen, Carlsbad, CA, USA).

According to the manufacturer’s manual, sequencing libraries of RNA-seq were performed at Beijing Genomics Institute (BGI, Shenzhen, China) using the Illumina Truseq RNA Sample Preparation Kit (Illumina, San Diego, USA). Briefly, polyadenylated RNAs were isolated using the Oligotex mRNA Midi Kit (Qiagen, Valencia, CA, USA). The RNA sequencing libraries were constructed using the SOLiD Whole Transcriptome Analysis Kit following the standard protocol (AB, Applied Biosystems, USA) and sequenced on the Applied Biosystems SOLiD platform to generate high-quality single-end reads. The raw reads were aligned to genome sequences, trimming off a nucleotide each from the 5′ and 3′ ends and allowing up to two mismatches. Reads mapped to multiple locations were discarded and only uniquely mapped reads were used for the subsequent analysis. Gene expression levels were measured in reads per kilobase of exon model per million mapped reads (RPKM).

To increase the power to detect biologically meaningful functions, a relatively relaxed criterion of fold change (2-fold) and *P*-value (<0.05) was used to filter differentially expressed mitochondria-related genes (DEGs) for biological analysis. The Database for Annotation, Visualization and Integrated Discovery (DAVID v6.7; http://david.abcc.ncifcrf.gov) was used to annotate biological themes (Gene Ontology, GO). The Kyoto Encyclopedia of Genes and Genomes (KEGG; http://www.genome.jp/kegg/) was used to determine the associated pathways. Phenotype annotations of differentially expressed genes were analyzed based on the Mouse Genome Informatics (MGI; http://www.informatics.jax.org/phenotypes.shtml) (MGI; http://www.informatics.jax.org/phenotypes.shtml) database.

### Selection of reference and target genes and primer design

The RNA extraction and RT-qPCR protocols were adapted from a previously published method [49]. Briefly, total RNA was extracted using Trizol (Invitrogen, Carlsbad, CA, USA) in accordance with the manufacturer’s protocol. The RNA concentration was measured using a NanoDrop ND-1000 spectrophotometer (Agilent Technologies, Palo Alto, CA, USA), and the purity of total RNA was then determined by the A260:280 and A260:230 ratios. For each sample, 1.00 μg of total RNA was reverse transcribed for complementary DNA (cDNA) synthesis using a PrimeScriptTM RT Reagent Kit with a gDNA Eraser (Takara Bio, Inc., Otsu, Japan) according to the manufacturer’s protocol. Next, RT-qPCR reactions were performed using Power SYBR green PCR master mix (Applied Biosystems, Carlsbad, CA) and a Q3 instrument (Applied Biosystems, CA, USA). The cycling program was 95°C (5 min) followed by 40 cycles of 95°C (10 s), 60°C (20 s), and 72°C (20 s). All reactions in this study were performed in duplicates for each cDNA sample. Three well-known porcine candidate reference genes and target genes were selected using the GenBank database and according to a literature review of several of the reference genes (Supplemental Table S7).

### Assessment of intestinal morphology

The samples of the jejunum, ileum, and colon were prepared according to a previously published method [50]. Briefly, the fixed samples were embedded in paraffin, and approximately 4.50-μm sections were mounted on poly-Lys-coated slides, deparaffinized, and rehydrated. The slides from each sample were stained with hematoxylin and eosin (HE) to examine morphology. Whole slide images were scanned by Mito More Than Microscopy, and 10 villi or crypts per sample were then selected to assess the intestinal architecture using the Motic Images Advanced 3.2 program (Motic China Group, Co., China). Then, the villus height: crypt depth ratio (IVR) was calculated.

### Periodic acid–Schiff (PAS) staining analysis

PAS staining was used to detect goblet cells in the intestine, including jejunum, ileum, and colon, as before [51]. Briefly, sections of the intestine were collected and immediately fixed in methanol-Carnoy’s fixative at 4°C for 2 h and then transferred to 100% ethanol. Fixed intestinal tissues were embedded in paraffin. Sections of 5-μm thickness were deparaffinized and then stained with PAS. Images were captured using an AxioScope A1 Zeiss microscope (Carl Zeiss) and analyzed using an image analysis system. Goblet cell counts and crypt depth measurements were determined from 10 well-oriented crypts.

### Transmission electron microscope (TEM)

Tissues from the immune system (e.g., liver, spleen, thymus, and inguinal lymph nodes) and reproductive system (e.g., uterus and ovaries) of pigs were sampled. And, the tissues were postfixed in 2.5% glutaraldehyde in cacodylate buffer (pH 7.2) overnight at 4°C. The pancreases were dissected, immersion-fixed with 4% paraformaldehyde for 8 h, and the sagittally sectioned at 100-mm intervals using a vibratome. Sections were incubated in 1% osmium tetroxide for 1 h, washed in 0.1 M phosphate buffer, and dehydrated via an ascending series of ethanol and propylene oxide and embedded in Epon. Ultrathin sections (70 nm) were cut and stained with uranyl acetate and lead citrate. The sections were imaged under a Philips CM120 scanning transmission electron microscope.

### Detection of zearalenone/its metabolites residues using UPLC-MS/MS

Detection of zearalenone/its metabolites residues from tissues and blood was conducted at Pribolab Pte. Ltd (Qingdao, China). Briefly, a 1 g (accurate to 0.0001 g) sample (or 1.00 mL blood) from each group was placed into a 50-mL centrifuge tube, and then 10 mL of acetic acid-sodium acetate buffer solution (0.05 mol/L, pH = 5.0) and 5000 U β-Glucuronidase buffer solution was also added into the tube. After mixing, this was hatched in an incubator at 37 C for 12 h. Afterwards, 10 mL acetonitrile and 200 μl of 1.0 mol/L NaOH solution were also added into the mixed buffer, and then shaken for 30 min, centrifuged at above 5000*g*/min at room temperature (20–25 C) for 3 min. Next, 10 mL supernatant was transferred to a new 50-mL centrifuge tube and was then diluted with 30 mL of 1 × PBS buffer. Eventually, pH was adjusted to 7.40 in the mixed buffer with 1.0 mol/L NaOH solution and was filtered using a glass fiber filter paper and then a 0.22-μm-pore-size membrane filter (Jinlong Co., Ltd., Tianjin, China).

For the HPLC-MS/MS procedure, separation was carried out in a Hypersil Gold AQ C_18_ column (100 mm×2.1 mm, 3 μm particle size; Thermo Scientific, Waltham MA, USA) using a TSQ QuantisTM triple-stage quadrupole mass spectrometer (Thermo Scientific, Waltham, MA, USA), and the column temperature was 30 °C. Chromatographic elution of zearalenone (ZEN) and its metabolites [Zearalanone (ZAN)], α-Zearalenol, β-Zearalenol, α-Zeranol, β-Zeranol] was performed with a mobile phase consisting of acetonitrile (part A) and ultrapure water (part B). For example, with respect to the separation ZEN and ZAN, an isocratic elution at a 0.4 mL/min flow rate started at 25% B and was held for 1 min. Next, 75% A at a 0.4 mL/min flow rate was started at 1 min and held for 6 min. Then, 40% B at a 0.4 mL/min flow rate was started at 6 min and held for 9 min. The injection volume was 5 μL. The mass spectrometer was operated using a turbo ion-spray ionization source configured for electrospray ionization (ESI) in positive and negative switching ion modes, and the acquisition was conducted using multiple reaction monitoring with a dwell time of 100 ms. The ion voltages for the positive and negative ion modes were both 3500 V. Resolutions at Q1 and Q3 were set to units. Argon gas (99% purity) was used in the ESI source and the collision cell. Optimal collision energies (CEs) and cone voltages were selected for each transition and retention time.

### Determination of plasma pro-inflammatory cytokines and LPS

Blood samples were allowed to clot at 4°C and centrifuged at 4000×*g* for 10 min to obtain plasma. The plasma samples were stored at −80°C until they were analyzed. The concentrations of plasma pro-inflammatory cytokines, including IL-1a, IL-1β, IFN-γ, IL-6, and IL-12, were detected using commercially available ELISA kits (Nanjing Jiancheng Bioengineering Institute, Nanjing, China) according to the manufacturer’s recommendations. Also, the concentration of plasma TNF-α was analyzed using available ELISA kits according to the manufacturer’s instruction (Sigma Aldrich, Cat# RAB0478-1KT, St. Louis, US). Additionally, the concentration of plasma lipopolysaccharides (LPS) was measured by a LPS ELISA kit according to the manufacturer’s instruction (CSB-E13066m, Cusabio, Wuhan, China).

### Western blot analysis

The procedure of Western blot was performed as described earlier [44]. Briefly, after protein samples were extracted from tissues, the concentration of protein was determined spectrophotometrically at OD_562_ using the Protein Quantitative Reagent Kit-BCA Method (ComWin Biotech, Co., Beijing, China). All antibodies are listed in Supplemental Table S6. GAPDH and/or tubulin were used as the loading Ctrl, and the normalization and quantification of the bands were carried out using Quantity-One software (Bio-Rad).

### In vitro experiment

#### Cell culture

The porcine small intestinal epithelial cells, IPEC-J2, were cultured in DMEM/F12 medium with 10% fetal bovine serum and 1% penicillin-streptomycin at 37 C in a humidified atmosphere of 5% CO_2_. For cell growth, culture medium was refreshed daily.

#### Zearalenone (ZEN) and sodium butyrate treatment

Cells (10,000/cm^2^) were plated into Petri dishes or well plates 24 h before ZEN and sodium butyrate treatment. To investigate the effects of ZEN and butyrate on the IPEC-J2, cells were exposed upon 60 μM zearalenone and/or supplemented with 5 mM sodium butyrate (Selleck, s1999) for 24 h. Afterwards, cell lysates from the three groups, including the control (Ctrl) group, the ZEN group, and the ZEN group supplemented with sodium butyrate (ZEN+Butyrate) were collected according to further purposes. All experiments were replicated at least three times.

For RT-qPCR in IPEC-J2 cells, it was performed as previously described (see the “Selection of reference and target genes and primer design” section). Primers used in this study are exhibited in Supplemental Table S7.

For Western blot in IPEC-J2 cells, it was also conducted as previously described (see the “Western blot analysis” section). All antibodies are listed in Supplemental Table S6. Tubulin was used as the loading control, and the normalization and quantification of the bands were carried out using Quantity-One software (Bio-Rad). For immunofluorescence staining (IF), after treatment cells were fixed with 4% paraformaldehyde at room temperature for 10 min and rinsed by 1×PBS three times. And then, 1% bovine serum albumin was used to block non-specific antigens for 30 min under thermoneutral conditions. Primary antibodies encompassing occludin (Proteintech, 27260-1-AP) and claudin-1 (Beyotime, AF6504) were diluted by 1×PBS at 1/500 dilutions, and cells were then incubated at 4 C overnight. Cells were washed by 1×PBS three times and secondary antibody Goat anti-Rabbit IgG (H+L) Fluor594-conjugated (Affinity, S0006) was diluted (1: 2,000), and they were then added into each well correspondingly. After 1 h of incubation, cells were washed by 1×PBS three times and photographed by a fluorescence microscope.

For reactive oxygen species (ROS) assay, cell lysates were collected and then detected by ROS (Beyotime, S0033S) assay kit in accordance with the manufacturer’s protocols.

For the TEM analysis in IPEC-J2 cells, it was performed as previously described (see the “Transmission electron microscope (TEM)” section).

### Statistical analysis

In this study, statistical analyses on the parameters were performed using IBM SPSS Statistics 23 (SPSS Inc., Chicago, IL) and GraphPad 8.0 PRISM®. Briefly, both in vitro and in vivo parameters were compared using a one-way ANOVA followed by Duncan’s post hoc tests (among groups). Considering Spearman’s correlation test, the relationship between microbial metabolites (e.g., SCFAs and LPS) and the core bacteria (at the family level) was also analyzed. For this, it first needed to confirm that each sample at each group was independent. Secondly, Shapiro–Wilk for normality test and Bartlett’s test for equal variances were then assessed, if the results were not in accordance with both the normal distribution and equal variances, non-parametric analyses (Kruskal–Wallis test) were then applied in this study. All data are presented as mean ± SEM. *P*-values <0.05 are considered statistically significant.

## Supplementary Information

**Additional file 1: Supplemental Table S1.** The levels of ZEN and it’s metabolites in both pre-starter (phase 1) and starter (phase 2) pigs.

**Additional file 2: Supplemental Table S2.** The health and growth of both pre-starter (phase 1) and starter (phase 2) pigs.

**Additional file 3: Supplemental Fig. S1.** (Related to Fig. 1c-d). Mycotoxin-contaminated food causes a larger vulvar area of pre-starter (phase 1; a) and starter (phase 2; b) between the control group and the mycotoxin (zearalenone, ZEN) group (*n*=4).

**Additional file 4: Supp Table S3-1.** List of differentially expressed genes (DEGs) between control (Ctrl) and zearalenone (ZEN) treatment group in pre-starter pig’s uterus, thymus, spleen, liver and inguinal lymph nodes. **Supp Table S3-2.** List of DEGs between Ctrl and ZEN treatment group in starter pig’s uterus, thymus, spleen, liver, inguinal lymph nodes and ovary. **Supp Table S3-3.** GO classification and enrichment of overlapping up-regulated genes between pre-starter and starter pig’s uterus. **Supp Table S3-4.** GO classification and enrichment of overlapping down-regulated genes between pre-starter and starter pig’s uterus. **Supp Table S3-5.** MGI phenotypes and Human diseases of genes (UP and DOWN) enriched by GO enrichment in pre-starter pig’s uterus. **Supp Table S3-6.** KEGG pathway enrichment of DEGs between Ctrl and ZEN treatment group in pre-starter pig’s uterus, thymus, spleen, liver and inguinal lymph nodes. **Supp Table S3-7.** KEGG pathway enrichment of DEGs between Ctrl and ZEN treatment group in starter pig’s uterus, thymus, spleen, liver and inguinal lymph nodes. **Supp Table S3-8.** List of DEGs involving immune diseases & system on the top 30 KEGG pathways in pre-starter and starter pig’s uterus. **Supp Table S3-9.** List of DEGs enriched in each overlapping KEGG pathways (top 30) between pre-starter and starter pig’s thymus, spleen, liver and inguinal lymph nodes. **Supp Table S3-10.** KEGG pathway enrichment of overlapping up-regulated genes in pre-starter and starter pig’s uterus. **Supp Table S3-11.** KEGG pathway enrichment of overlapping down-regulated genes in pre-starter and starter pig’s uterus. **Supp Table S3-12.** GO enrichment of DEGs between Ctrl and ZEN treatment group in pre-starter and starter pig’s uterus. **Supp Table S3-13.** List of genes for expression pattern analysis in starter pig’s uterus and thymus. **Supp Table S3-14.** KEGG pathway enrichment of genes enriched in profile 2 of expression pattern analysis in starter pig’s uterus and thymus. **Supp Table S3-15.** GO enrichment of genes enriched in profile 2 of expression pattern analysis in starter pig’s uterus and thymus. **Supp Table S3-16.** GO enrichment of up-regulated and down-regulated genes between Ctrl and ZEN treatment group in starter pig’s ovary. **Supp Table S3-17.** GO classification and enrichment of DEGs between Ctrl and ZEN treatment group in starter pig’s overy.

**Additional file 5: Supplemental Fig. S2.** RNA-seq analysis unravels mycotoxin-induced toxicity on reproductive system (ovary).

**Additional file 6: Supplemental Fig. S3.** (Related to Fig. 4c-d). OTU based PLS-DA score plots of five gut sections (duodenum, jejunum, ileum, caecum, colon) in pre-starter (a) and starter (b) pigs between the Ctrl group and the ZEN group (*n*=8).

**Additional file 7: Supplemental Fig. S4.** (Related to Fig. 4e-f). Bacterial compositions at the genus level and its dominant bacterial genera (relative abundance > 1%) of five gut sections (duodenum, jejunum, ileum, caecum, colon) of pre-starter (a) and starter pigs (b) that exposed to ZEN (*n*=8). Bar values are means ± SEM. *P < 0.05.

**Additional file 8: Supplemental Fig. S5.** (Related to Fig. 6a). Identification of recombinant *B. subtilis*-expressing* ZLHY-6* (the ZEN degrading enzyme) (Bs-Z6) and its fermentation conditions.

**Additional file 9: Supplemental Fig. S6.** (Related to Fig. 6d). During phase 3, bacterial compositions at the genus level and its dominant bacterial genera (relative abundance > 1%) of the colon of pigs among the control (Ctrl), zearalenone (ZEN), ZEN supplemented with recombinant Bs-Z6 strain (Bs-Z6) groups (*n*=8). Bar values are means ± SEM. *P < 0.05.

**Additional file 10: Supplemental Table S4.** The levels of ZEN and it’s metabolites in pigs during phase 3.

**Additional file 11: Supplemental Table S5.** The health and growth of pigs during phase 3.

**Additional file 12: Supplemental Fig. S7.** (Related to Fig. 7a). During phase 3, modified microbial metabolites by recombinant Bs-Z6 strain rescue the vulvar area of pigs that expose to ZEN (*n*=4). Bar values are means ± SEM. *P < 0.05.

**Additional file 13: Supplemental Fig. S8.** (Related to Fig. 7e-j). During phase 3, modified microbial metabolites by recombinant Bs-Z6 strain corrected gene expression patterns and functions of reproductive organs (e.g., uterus) and immune organs (e.g., thymus) in pigs that exposed to ZEN (*n*=4).

**Additional file 14: Supplemental Table S6.** Key resources table.

**Additional file 15: Supplemental Table S7.** List of primers used in RT-qPCR.

## Data Availability

All source data and materials are provided with this paper. Additional data that support the findings of this study are available from the corresponding authors upon reasonable request.

## Abbreviations

ZEN: Zearalenone
α-ZOL: α-Zearalenol
β-ZOL: β-Zearalenol
α-ZAL: α-Zeranol
β-ZAL: β-Zeranol
ZAN: Zearalanone
SCFAs: Short-chain fatty acids
LPS: Lipopolysaccharides
Bs-Z6 strain: Recombinant *Bacillus subtilis* 168-expressing ZEN-degrading enzyme ZLHY-6
EV: Empty vector
IPEC-J2: Porcine small intestinal epithelial cell line
ESFA: European Food Safety Authority
AQSIQ: General Administration of Quality Supervision, Inspection and Quarantine of China
SAC: Standardization Administration of China
OTUs: Operational taxonomic units
PLS-DA: Partial least squares-discriminant analysis
GO: Gene ontology
MGI: Mouse Genome Informatics
KEGG: Kyoto Encyclopedia of Genes and Genomes
PAS: Periodic acid–Schiff
DEGs: Differentially expressed genes
ANOVA: Analysis of variance
PCR: Polymerase chain reaction
cDNA: Complementary DNA
LB: Luria-Bertani
HE: Hematoxylin and eosin
IVR: The villus height: crypt depth ratio
IF: Immunofluorescence staining
ROS: Reactive oxygen species
