# Supplementary material for: Zearalenone disturbs the reproductive-immune axis in pigs: the role of gut microbial metabolites
Source: Microbiome. 2022 Dec 19;10:234. doi: 10.1186/s40168-022-01397-7 (PMC9762105; doi:10.1186/s40168-022-01397-7)
Supplement: Supplementary file 2 — Additional file 1: Supplemental Table S1. The levels of ZEN and it’s metabolites in both pre-starter (phase 1) and starter (phase 2) pigs. [file 40168_2022_1397_MOESM1_ESM.docx]

**Supplemental Table S1. The levels of ZEN and it's metabolites in both pre-starter (phase 1) and starter (phase 2) pigs.**

| Phase-1 | ZEN | | | ZEN's metabolites | | | | | | | | | | | | | | |
| --- | --- | --- | --- | --- | --- | --- | --- | --- | --- | --- | --- | --- | --- | --- | --- | --- | --- | --- |
|  |  |  |  | **α-ZOL** | | | **β-ZOL** | | | **α-ZAL** | | | **β-ZAL** | | |  | **ZAN** | |
|  | Ctrl | ZEN | *P*-value | Ctrl | ZEN | *P*-value | Ctrl | ZEN | *P*-value | Ctrl | ZEN | *P*-value | Ctrl | ZEN | *P*-value | Ctrl | ZEN | *P*-value |
| Duodenum | 18.86^a^ | 1072.17 | <0.01 | 18.47 | 1095.63 | <0.01 | 2.11 | 22.61 | <0.01 | 0.79 | 2.02 | <0.01 | ND | 0.80 | - | 0.96 | 10.16 | <0.01 |
| Jejunum | 16.18 | 637.77 | <0.01 | 15.54 | 725.89 | <0.01 | 1.38 | 23.74 | <0.01 | 0.53 | 1.99 | <0.01 | ND | 1.20 | - | 1.31 | 12.58 | <0.01 |
| Ileum | 19.92 | 778.21 | <0.01 | 16.72 | 833.84 | <0.01 | 1.58 | 41.27 | <0.01 | 0.30 | 2.11 | <0.01 | ND | 1.77 | - | 1.31 | 9.77 | <0.01 |
| Caecum | 13.99 | 536.40 | <0.01 | 15.19 | 638.78 | <0.01 | 1.40 | 29.61 | <0.01 | ND | 1.67 | - | ND | 0.33 | - | 0.68 | 8.72 | <0.01 |
| Colon | 14.28 | 1008.57 | <0.01 | 17.17 | 1262.66 | <0.01 | 2.18 | 56.07 | <0.01 | 0.72 | 4.56 | <0.01 | ND | 0.91 | - | 0.96 | 17.18 | <0.01 |
| Liver | 0.55 | 14.02 | <0.01 | 0.90 | 28.17 | <0.01 | ND | 2.99 | - | ND | ND | - | ND | ND | - | ND | ND | - |
| Thymus | 0.69 | 5.49 | <0.01 | 0.64 | 5.27 | <0.01 | 0.65 | 1.74 | <0.01 | ND | ND | - | ND | ND | - | ND | 0.67 | - |
| Spleen | 0.64 | 6.36 | <0.01 | 0.90 | 6.77 | <0.01 | ND | 2.99 | - | ND | ND | - | ND | ND | - | ND | ND | - |
| Inguinal lymph node | 0.48 | 3.43 | <0.01 | 0.59 | 2.18 | <0.01 | ND | 1.83 | - | ND | ND | - | ND | ND | - | ND | 0.44 | - |
| Uterus | 0.49 | 10.27 | <0.01 | ND | 5.70 | - | ND | 1.20 | - | ND | ND | - | ND | ND | - | ND | 0.66 | - |
| Ovary | 0.61 | 8.94 | <0.01 | 0.61 | 7.51 | <0.01 | 0.32 | 2.10 | - | ND | ND | - | ND | ND | - | ND | ND | - |
| Blood | 0.22 | 3.37 | <0.01 | ND | 0.74 | - | ND | ND | - | ND | ND | - | ND | ND | - | ND | ND | - |
| Phase-2 | **ZEN** | | | **ZEN's metabolites** | | | | | | | | | | | | | | |
|  |  |  |  | **α-ZOL** | | | **β-ZOL** | | | **α-ZAL** | | | **β-ZAL** | | |  | **ZAN** | |
|  | Ctrl | ZEN | *P*-value | Ctrl | ZEN | *P*-value | Ctrl | ZEN | *P*-value | Ctrl | ZEN | *P*-value | Ctrl | ZEN | *P*-value | Ctrl | ZEN | *P*-value |
| Duodenum | 24.54 | 1187.79 | <0.01 | 20.06 | 994.21 | <0.01 | 2.17 | 34.71 | <0.01 | 0.64 | 3.13 | <0.01 | ND | 0.90 | - | 1.51 | 8.61 | <0.01 |
| Jejunum | 19.20 | 1049.90 | <0.01 | 13.57 | 1030.03 | <0.01 | 1.93 | 44.88 | <0.01 | 0.44 | 3.39 | <0.01 | ND | 1.20 | - | 1.48 | 13.53 | <0.01 |
| Ileum | 16.31 | 1086.36 | <0.01 | 16.49 | 945.54 | <0.01 | 1.59 | 44.22 | <0.01 | 0.58 | 3.12 | <0.01 | ND | 1.14 | - | 1.34 | 12.42 | <0.01 |
| Caecum | 23.44 | 856.83 | <0.01 | 19.66 | 839.06 | <0.01 | 1.98 | 51.74 | <0.01 | 0.42 | 3.98 | <0.01 | ND | 0.64 | - | 1.88 | 14.80 | <0.01 |
| Colon | 23.64 | 722.96 | <0.01 | 18.21 | 747.18 | <0.01 | 1.93 | 43.04 | <0.01 | 0.81 | 3.41 | <0.01 | ND | 0.69 | - | 1.63 | 13.77 | <0.01 |
| Liver | 0.65 | 11.74 | <0.01 | 0.93 | 15.47 | <0.01 | 0.32 | 3.01 | <0.01 | ND | 0.68 | - | ND | 0.26 | - | ND | 0.58 | - |
| Thymus | 0.42 | 5.24 | <0.01 | 0.65 | 4.80 | <0.01 | ND | 2.04 | - | ND | ND | - | ND | ND | - | ND | 0.57 | - |
| Spleen | 0.82 | 6.12 | <0.01 | 0.73 | 6.47 | <0.01 | 0.37 | 2.36 | <0.01 | ND | ND | - | ND | ND | - | ND | 0.51 | - |
| Inguinal lymph node | 0.56 | 4.20 | <0.01 | 0.56 | 2.68 | <0.01 | ND | 0.77 | - | ND | ND | - | ND | ND | - | ND | ND | - |
| Uterus | 0.44 | 11.93 | <0.01 | 0.31 | 8.99 | <0.01 | ND | 1.57 | - | ND | ND | - | ND | ND | - | ND | ND | - |
| Ovary | 0.71 | 9.66 | <0.01 | 0.48 | 9.20 | <0.01 | 0.15 | 1.30 | <0.01 | ND | 0.85 | - | ND | ND | - | ND | 0.46 | - |
| Blood | 0.30 | 5.45 | <0.01 | ND | 0.69 | - | ND | 0.62 | - | ND | ND | - | ND | ND | - | ND | ND | - |

**Note:** Zearalenone=ZEN; α-Zearalenol=α-ZOL; β-Zearalenol=β-ZOL; α-Zeranol=α-ZAL; β-Zeranol=β-ZAL; Zearalanone=ZAN.

^a,b^ superscripts are not displayed in the same row in this table, please see *P*-values. Values are means, n = 8 (each group); *P* < 0.05 significant at the 0.05% level.
