# Supplementary material for: Zearalenone disturbs the reproductive-immune axis in pigs: the role of gut microbial metabolites
Source: Microbiome. 2022 Dec 19;10:234. doi: 10.1186/s40168-022-01397-7 (PMC9762105; doi:10.1186/s40168-022-01397-7)
Supplement: Supplementary file 3 — Additional file 2: Supplemental Table S2. The health and growth of both pre-starter (phase 1) and starter (phase 2) pigs. [file 40168_2022_1397_MOESM2_ESM.docx]

**Supplemental Table S2. The health and growth of both pre-starter (phase 1) and starter (phase 2) pigs.**

| Items | Phase 1 | | *P*-values |
| --- | --- | --- | --- |
|  | Ctrl | ZEN |  |
| Initial body weight(kg) | 8.67±0.10 | 9.00±0.34 | 0.38 |
| Final body weight(kg) | 12.87±0.28 | 13.73±0.44 | 0.15 |
| Average daily gain(g) | 300±16.50 | 338±23.71 | 0.24 |
| Average daily feed intake(g) | 518±16.57 | 533±21.58 | 0.60 |
| Feed-to-gain ratio | 1.74±0.11 | 1.59±0.07 | 0.29 |
| Items | **Phase 2** | | *P*-values |
|  | Ctrl | ZEN |  |
| Initial body weight(kg) | 20.00±0.41 | 20.08±0.42 | 0.90 |
| Final body weight(kg) | 29.17±0.73 | 29.33±0.40 | 0.85 |
| Average daily gain(g) | 611±33.33 | 606±16.67 | 0.89 |
| Average daily feed intake(g) | 1169±28.45 | 1171±23.24 | 0.96 |
| Feed-to-gain ratio | 1.93±0.08 | 1.94±0.08 | 0.90 |

**Note:** Values are means, n = 4 (pen is the experimental unit).

*P* < 0.05 significant at the 0.05% level.
