# Supplementary material for: Zearalenone disturbs the reproductive-immune axis in pigs: the role of gut microbial metabolites
Source: Microbiome. 2022 Dec 19;10:234. doi: 10.1186/s40168-022-01397-7 (PMC9762105; doi:10.1186/s40168-022-01397-7)
Supplement: Supplementary file 4 — Additional file 3: Supplemental Fig. S1. (Related to Fig. 1c-d). Mycotoxin-contaminated food causes a larger vulvar area of pre-starter (phase 1; a) and starter (phase 2; b) between the control group and the mycotoxin (zearalenone, ZEN) group (n=4). [file 40168_2022_1397_MOESM3_ESM.docx]

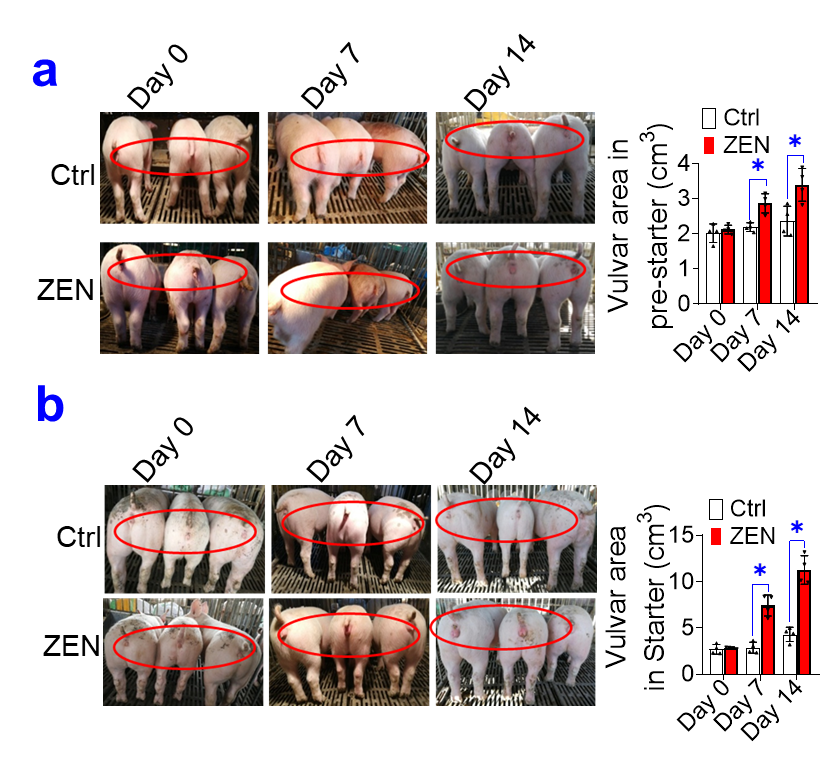


**Supplemental Fig. S1 (Related to Fig. 1c-d).** Mycotoxin-contaminated food causes a larger vulvar area of pre-starter (phase 1; **a**) and starter (phase 2; **b**) between the control group and the mycotoxin (zearalenone, ZEN) group (n=4).

Bar values are means ± SEM. ******P* < 0.05.
