# Supplementary material for: Zearalenone disturbs the reproductive-immune axis in pigs: the role of gut microbial metabolites
Source: Microbiome. 2022 Dec 19;10:234. doi: 10.1186/s40168-022-01397-7 (PMC9762105; doi:10.1186/s40168-022-01397-7)
Supplement: Supplementary file 6 — Additional file 5: Supplemental Fig. S2. RNA-seq analysis unravels mycotoxin-induced toxicity on reproductive system (ovary). [file 40168_2022_1397_MOESM5_ESM.docx]

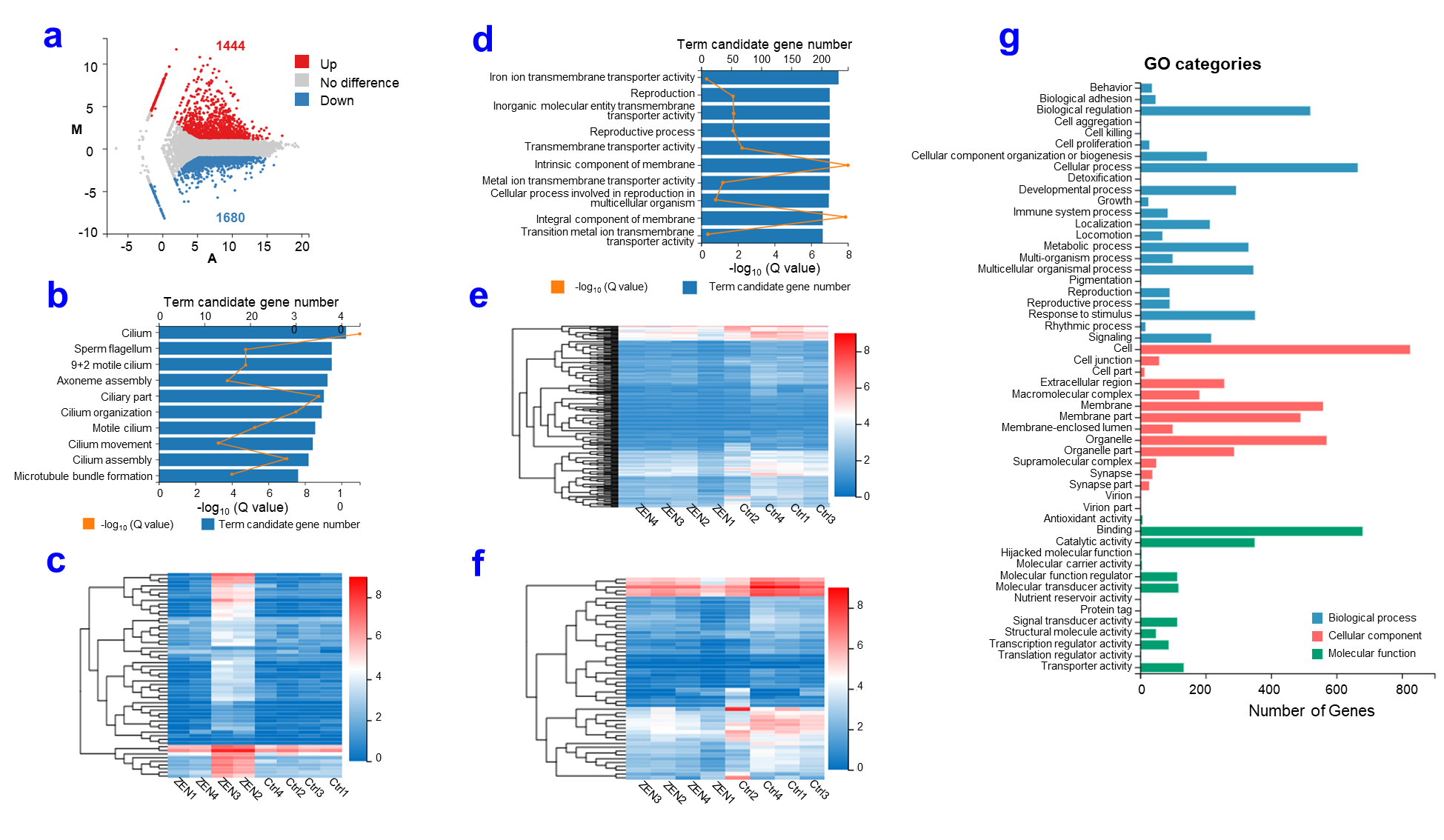


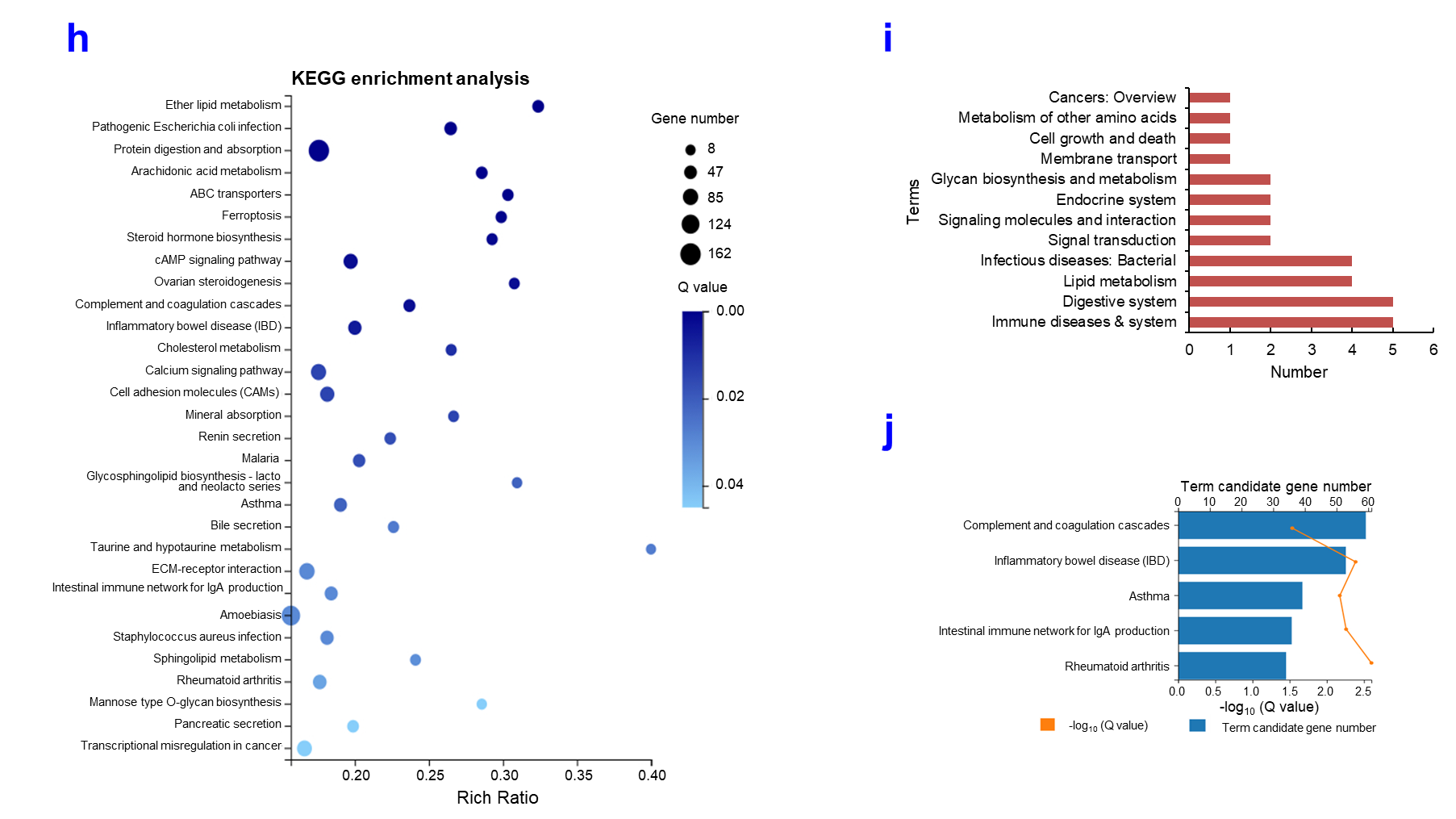
 **Supplemental Fig. S2.** RNA-seq analysis unravels mycotoxin-induced toxicity on reproductive system (ovary).

**a,** MA plot of different expression genes (DEGs) in the ovary of starter pig between the Ctrl group and the ZEN group (n=4). **b,** Top 10 GO enrichment terms of up-regulated DEGs. **c,** Heatmap of genes enriched in top 10 GO enrichment terms of up-regulated DEGs. **d,** Top 10 GO enrichment terms of down-regulated DEGs; **e,** Heatmap of seven top genes enriched GO enrichment terms of down-regulated DEGs that related to ion transport and membrane structure. **f,** Heatmap of three top genes enriched GO enrichment terms of down-regulated DEGs that related to reproduction. **g,** GO classification of DEGs. **h,** Top30 KEGG pathways of DEGs; **i,** Classification of pathway enrichment terms according to top 30 KEGG pathways. **j,** Pathways involved in immune disease & system according to top 30 KEGG pathways.
