# Supplementary material for: Zearalenone disturbs the reproductive-immune axis in pigs: the role of gut microbial metabolites
Source: Microbiome. 2022 Dec 19;10:234. doi: 10.1186/s40168-022-01397-7 (PMC9762105; doi:10.1186/s40168-022-01397-7)
Supplement: Supplementary file 7 — Additional file 6: Supplemental Fig. S3. (Related to Fig. 4c-d). OTU based PLS-DA score plots of five gut sections (duodenum, jejunum, ileum, caecum, colon) in pre-starter (a) and starter (b) pigs between the Ctrl group and the ZEN group (n=8). [file 40168_2022_1397_MOESM6_ESM.docx]

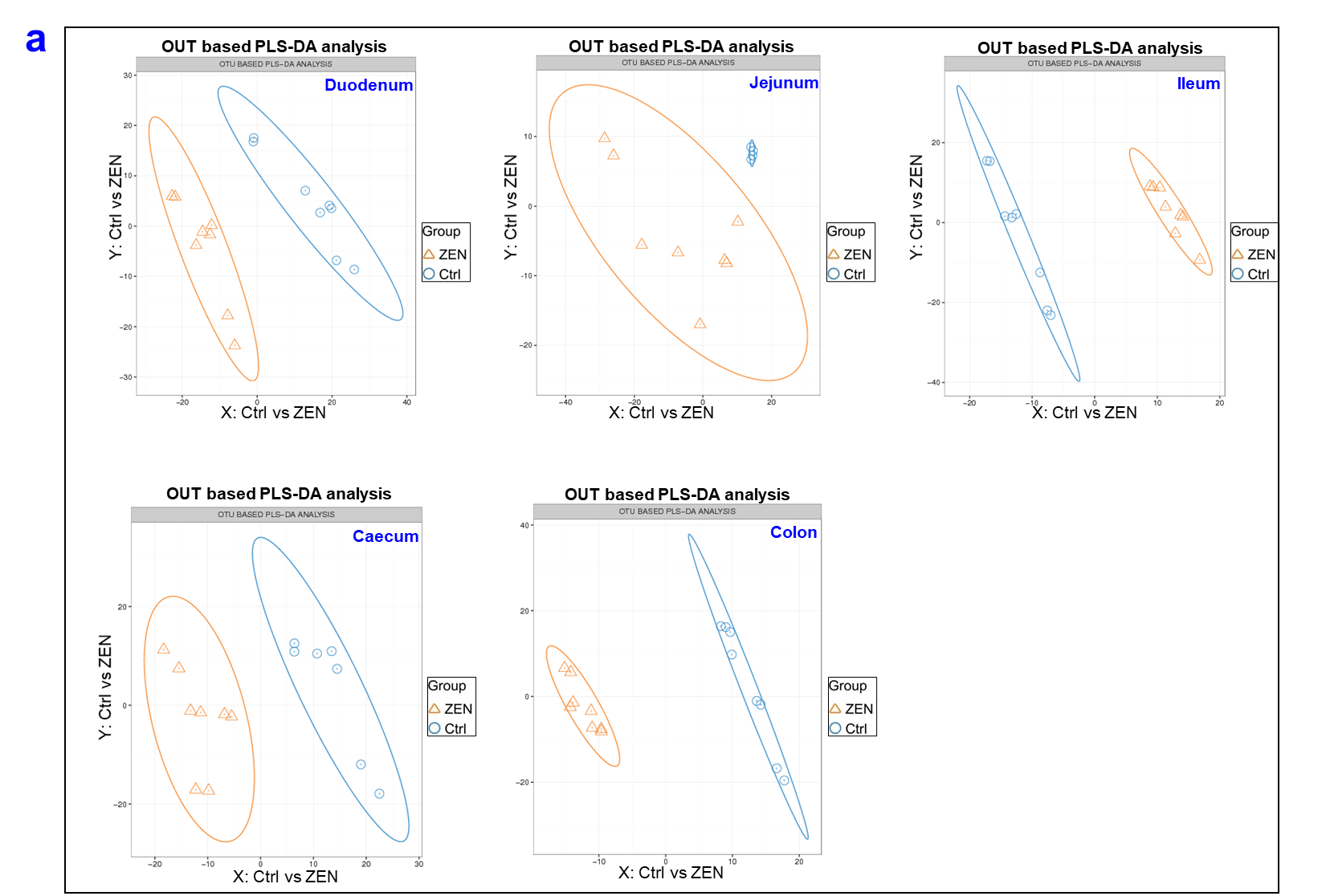


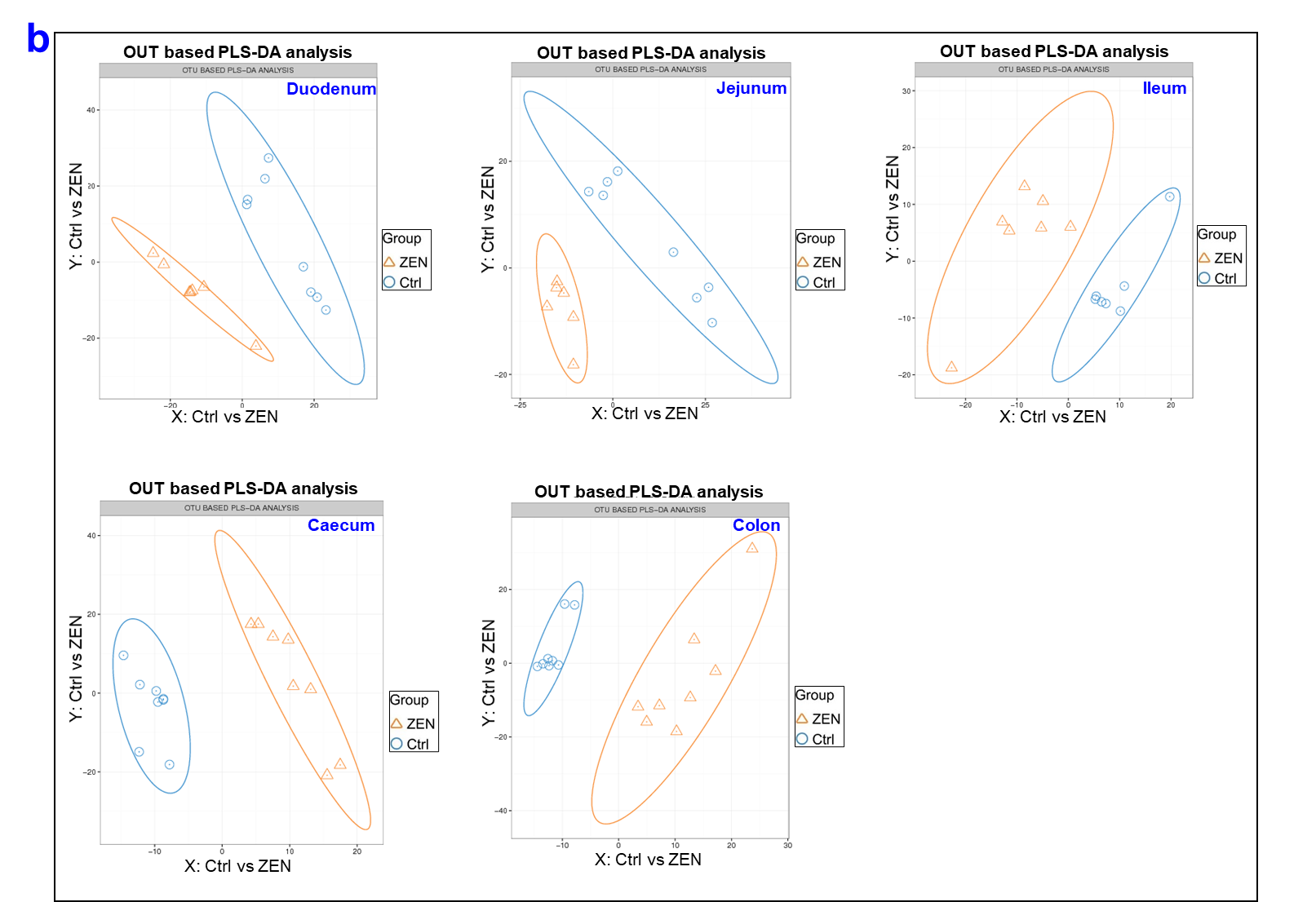


**Supplemental Fig. S3 (Related to Fig. 4c-d).** OTU based PLS-DA score plots of five gut sections (duodenum, jejunum, ileum, caecum, colon) in pre-starter (**a**) and starter (**b**) pigs between the Ctrl group and the ZEN group (n=8).
