# Supplementary material for: Zearalenone disturbs the reproductive-immune axis in pigs: the role of gut microbial metabolites
Source: Microbiome. 2022 Dec 19;10:234. doi: 10.1186/s40168-022-01397-7 (PMC9762105; doi:10.1186/s40168-022-01397-7)
Supplement: Supplementary file 8 — Additional file 7: Supplemental Fig. S4. (Related to Fig. 4e-f). Bacterial compositions at the genus level and its dominant bacterial genera (relative abundance > 1%) of five gut sections (duodenum, jejunum, ileum, caecum, colon) of pre-starter (a) and starter pigs (b) that exposed to ZEN (n=8). Bar values are means ± SEM. *P < 0.05. [file 40168_2022_1397_MOESM7_ESM.docx]

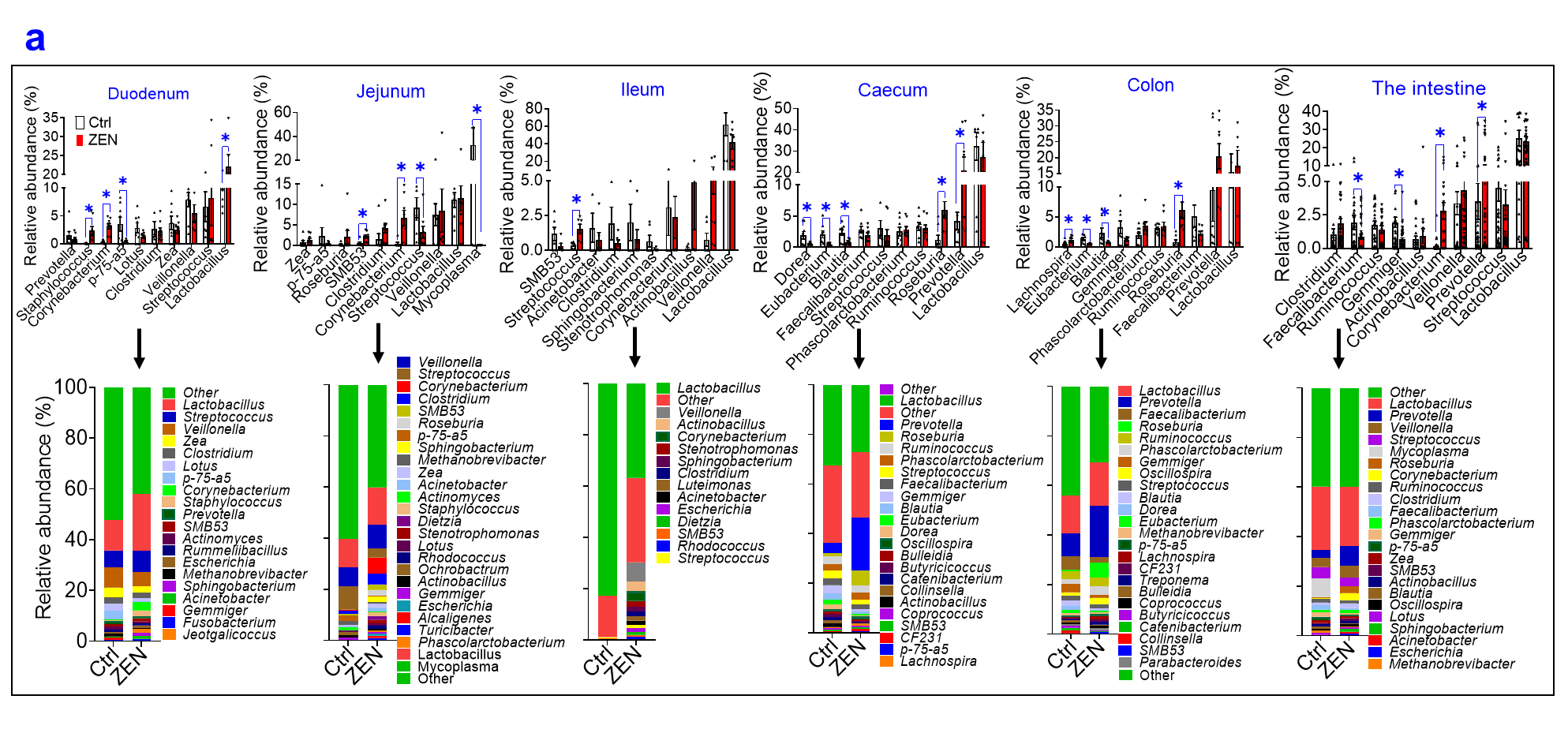

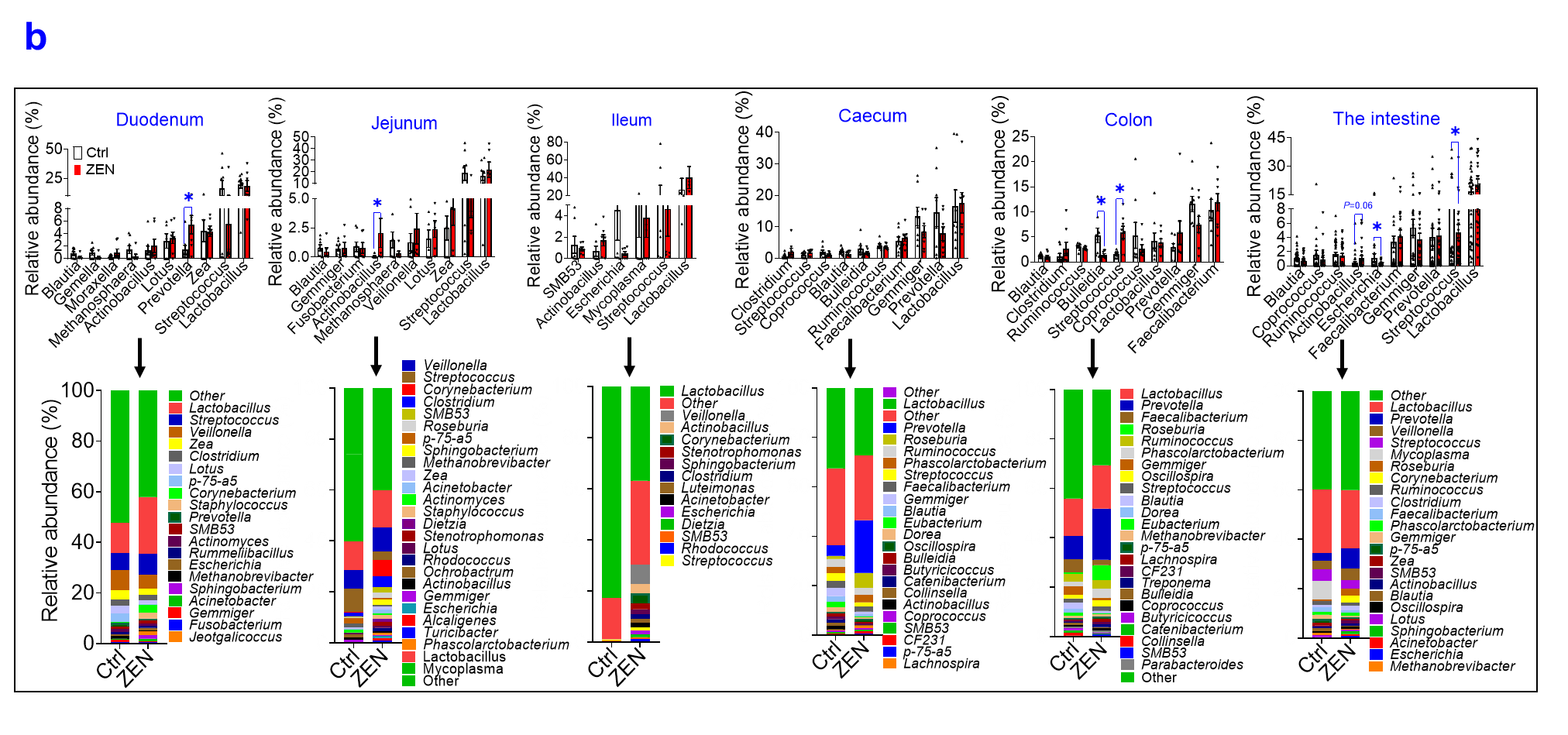
 **Supplemental Fig. S4 (Related to Fig. 4e-f).** Bacterial compositions at the genus level and its dominant bacterial genera (relative abundance > 1%) of five gut sections (duodenum, jejunum, ileum, caecum, colon) of pre-starter (**a**) and starter pigs (**b**) that exposed to ZEN (n=8). Bar values are means ± SEM. ******P* < 0.05.
