# Supplementary material for: Zearalenone disturbs the reproductive-immune axis in pigs: the role of gut microbial metabolites
Source: Microbiome. 2022 Dec 19;10:234. doi: 10.1186/s40168-022-01397-7 (PMC9762105; doi:10.1186/s40168-022-01397-7)
Supplement: Supplementary file 9 — Additional file 8: Supplemental Fig. S5. (Related to Fig. 6a). Identification of recombinant B. subtilis-expressing ZLHY-6 (the ZEN degrading enzyme) (Bs-Z6) and its fermentation conditions. [file 40168_2022_1397_MOESM8_ESM.docx]

**
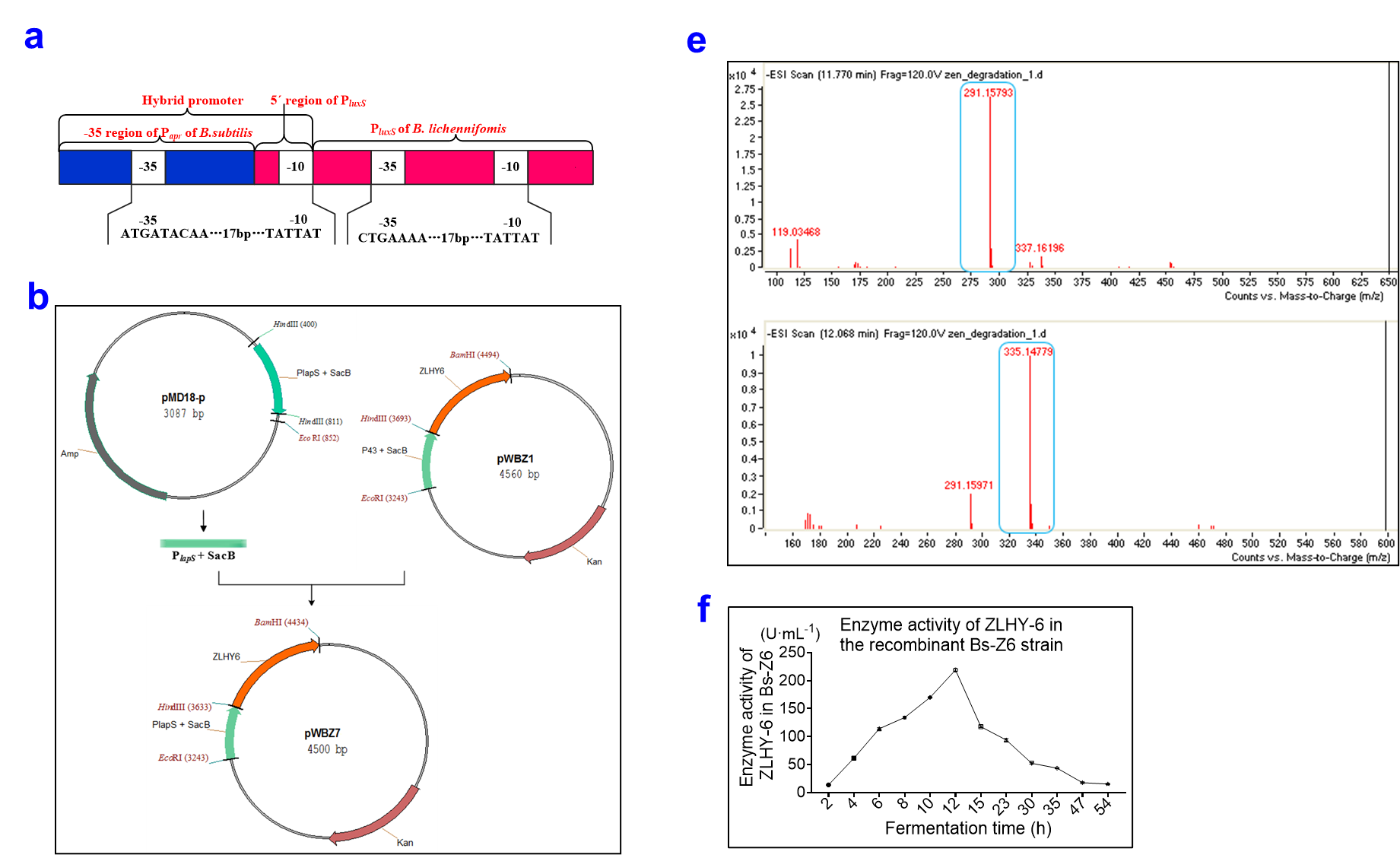

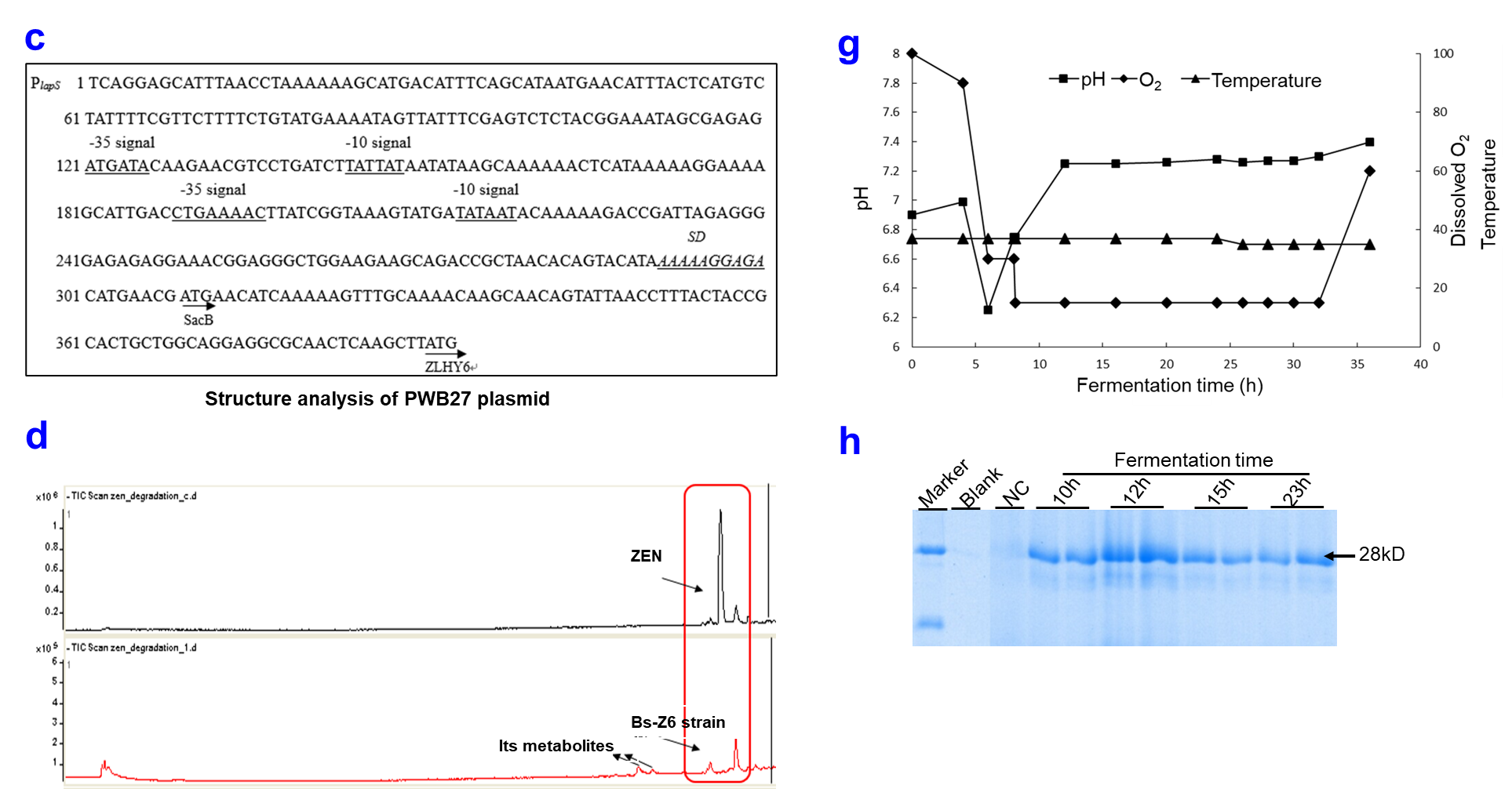
 Supplemental Fig. S5 (Related to Fig. 6a).** Identification of recombinant *B. subtilis*-expressing ZLHY-6 (the ZEN degrading enzyme) (Bs-Z6) and its fermentation conditions.

**a,** Structure of promoter *P_lapS_*. **b,** Construction schematic diagram of recombinant expression pWBZ7 plasmid. *ZLHY6*: ZEN degrading enzyme gene; *SacB*: signal peptide; pMD18-p: pMD18-T vector containing objective fragments. **c,** Structure analysis of recombinant expression pWBZ7 plasmid. The conservative regions of *P_lapS_* are underlined; SD regions are italics and underlined; Initiation codons of *SacB* and *ZLHY6* are arrowed. **d,** First level of mass spectrometry analysis of ZEN degradation products. **e,** Secondary level of mass spectrometry analysis of ZEN degradation products. **f,** Comparison of degradation enzyme *ZLHY6* activity of recombinant Bs-Z6 strain during the course of its fermentation (2, 4, 6, 8, 10, 12, 15, 23, 30, 35, 47, 54 hours). **g,** Regulation of fermentation conditions (e.g., dissolved O_2_, pH, and temperature) under different fermentation time (2, 4, 6, 8, 10, 12, 15, 23, 30, 35 hours). **h,** SDS-PAGE analysis for the fermentation supernatant from recombinant Bs-Z6 strain under different fermentation time. Lane marker: Protein ladder; Lane Blank: Blank control; Lane NC: Empty vector; Lane 1-8: the fermentation supernatant from recombinant Bs-Z6 strain at 10 h, 12 h,15 h, 23 h, respectively.
