# Supplementary material for: Zearalenone disturbs the reproductive-immune axis in pigs: the role of gut microbial metabolites
Source: Microbiome. 2022 Dec 19;10:234. doi: 10.1186/s40168-022-01397-7 (PMC9762105; doi:10.1186/s40168-022-01397-7)
Supplement: Supplementary file 10 — Additional file 9: Supplemental Fig. S6. (Related to Fig. 6d). During phase 3, bacterial compositions at the genus level and its dominant bacterial genera (relative abundance > 1%) of the colon of pigs among the control (Ctrl), zearalenone (ZEN), ZEN supplemented with recombinant Bs-Z6 strain (Bs-Z6) groups (n=8). Bar values are means ± SEM. *P < 0.05. [file 40168_2022_1397_MOESM9_ESM.docx]

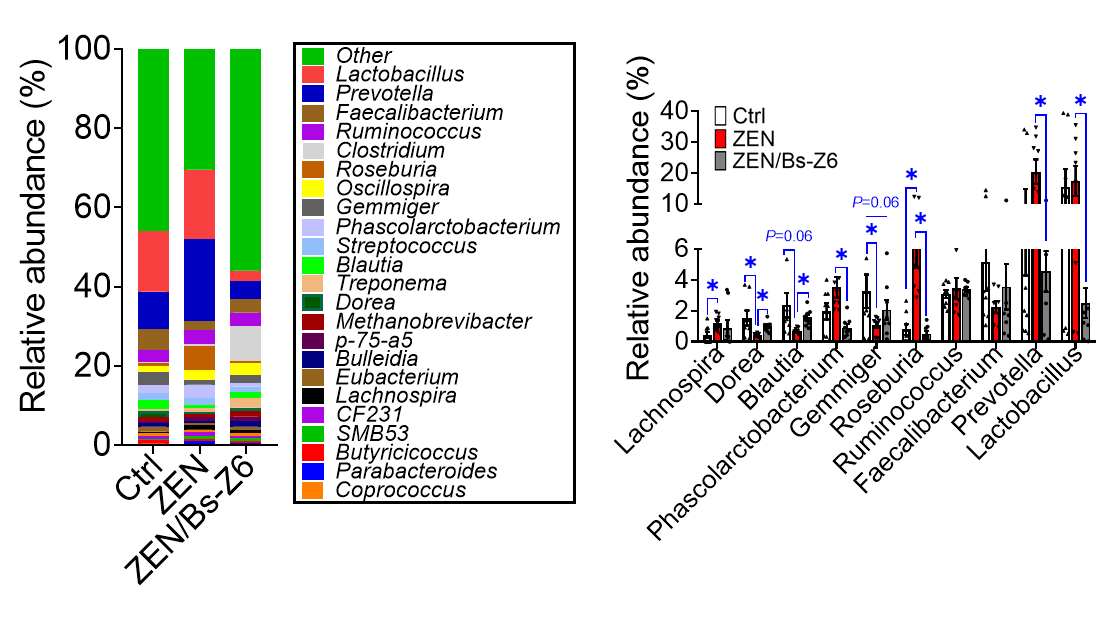
 **Supplemental Fig. S6 (Related to Fig. 6d).** During phase 3, bacterial compositions at the genus level and its dominant bacterial genera (relative abundance > 1%) of the colon of pigs among the control (Ctrl), zearalenone (ZEN), ZEN supplemented with recombinant Bs-Z6 strain (Bs-Z6) groups (n=8). Bar values are means ± SEM. ******P* < 0.05.
