# Supplementary material for: Zearalenone disturbs the reproductive-immune axis in pigs: the role of gut microbial metabolites
Source: Microbiome. 2022 Dec 19;10:234. doi: 10.1186/s40168-022-01397-7 (PMC9762105; doi:10.1186/s40168-022-01397-7)
Supplement: Supplementary file 11 — Additional file 10: Supplemental Table S4. The levels of ZEN and it’s metabolites in pigs during phase 3. [file 40168_2022_1397_MOESM10_ESM.docx]

**Supplemental Table S4. The levels of ZEN and it's metabolites in pigs during phase 3.**

|  | ZEN | | | | α-ZOL | | | | β-ZOL | | | |
| --- | --- | --- | --- | --- | --- | --- | --- | --- | --- | --- | --- | --- |
|  | Ctrl | ZEN | ZEN/Bs-Z6 | *P*-value | Ctrl | ZEN | ZEN/Bs-Z6 | *P*-value | Ctrl | ZEN | ZEN/Bs-Z6 | *P*-value |
| Colon | 22.29^a^ | 756.44^b^ | 78.40^c^ | <0.01 | 22.59^a^ | 694.10^b^ | 69.74^c^ | <0.01 | 2.38^a^ | 61.82^b^ | 7.92^c^ | <0.01 |
| Liver | 0.92^a^ | 12.01^b^ | 2.41^a^ | <0.01 | 1.06^a^ | 14.23^b^ | 2.04^a^ | <0.01 | 0.47^a^ | 3.71^b^ | 0.94^a^ | <0.01 |
| Uterus | 0.61^a^ | 8.89^b^ | 1.20^a^ | <0.01 | 0.45^a^ | 6.91^b^ | 1.33^c^ | <0.01 | 0.62^a^ | 1.70^b^ | 0.95^ab^ | <0.01 |
| Blood | 0.45^a^ | 7.63^b^ | 1.09^c^ | <0.01 | 0.37^a^ | 3.50^b^ | 0.79^a^ | <0.01 | ND | 0.76 | 0.56 | - |
|  | **α-ZAL** | | | | **β-ZAL** | | | | **ZAN** | | | |
|  | Ctrl | ZEN | ZEN/Bs-Z6 | *P*-value | Ctrl | ZEN | ZEN/Bs-Z6 | *P*-value | Ctrl | ZEN | ZEN/Bs-Z6 | *P*-value |
| Colon | 0.89^a^ | 4.57^b^ | 1.55^c^ | <0.01 | 0.32^a^ | 1.38^b^ | 0.88^ab^ | <0.01 | 1.73^a^ | 16.07^b^ | 2.85^a^ | <0.01 |
| Liver | ND | 0.99 | ND | - | ND | 0.65 | ND | - | ND | ND | ND | - |
| Uterus | ND | ND | ND | - | ND | ND | ND | - | ND | ND | ND | - |
| Blood | ND | ND | ND | - | ND | ND | ND | - | ND | ND | ND | - |

**Note:** Zearalenone=ZEN; Zearalanone=ZAN; α-Zearalenol=α-ZOL; β-Zearalenol=β-ZOL; α-Zeranol=α-ZAL; β-Zeranol=β-ZAL.

^a,b,c^ superscripts means within the same row without common superscripts differ significantly (*P* < 0.05).

Values are means, n =8 (each group). ND= no significance. *P* < 0.05 significant at the 0.05% level.
