# Supplementary material for: Zearalenone disturbs the reproductive-immune axis in pigs: the role of gut microbial metabolites
Source: Microbiome. 2022 Dec 19;10:234. doi: 10.1186/s40168-022-01397-7 (PMC9762105; doi:10.1186/s40168-022-01397-7)
Supplement: Supplementary file 12 — Additional file 11: Supplemental Table S5. The health and growth of pigs during phase 3. [file 40168_2022_1397_MOESM11_ESM.docx]

**Supplemental Table S5. The health and growth of pigs during phase 3.**

| Items | Phase 3 | | | *P*-values |
| --- | --- | --- | --- | --- |
|  | Ctrl | ZEN | ZEN/Bs-Z6 |  |
| Initial body weight(kg) | 63.99±1.32 | 62.33±1.82 | 65.00±3.08 | 0.66 |
| Final body weight(kg) | 102.00±5.06 | 96.94±4.87 | 101.67±7.47 | 0.74 |
| Average daily gain(g) | 895±138.61 | 824±126.13 | 873±119.54 | 0.89 |
| Average daily feed intake(g) | 2.05±0.17 | 1.87±0.25 | 2.39±0.13 | 0.28 |
| Feed-to-gain ratio | 2.51±0.50 | 2.32±0.28 | 2.83±0.23 | 0.58 |

**Note:** Values are means, n = 4 (pen is the experimental unit).

*P* < 0.05 significant at the 0.05% level.
