# Supplementary material for: Zearalenone disturbs the reproductive-immune axis in pigs: the role of gut microbial metabolites
Source: Microbiome. 2022 Dec 19;10:234. doi: 10.1186/s40168-022-01397-7 (PMC9762105; doi:10.1186/s40168-022-01397-7)
Supplement: Supplementary file 13 — Additional file 12: Supplemental Fig. S7. (Related to Fig. 7a). During phase 3, modified microbial metabolites by recombinant Bs-Z6 strain rescue the vulvar area of pigs that expose to ZEN (n=4). Bar values are means ± SEM. *P < 0.05. [file 40168_2022_1397_MOESM12_ESM.docx]

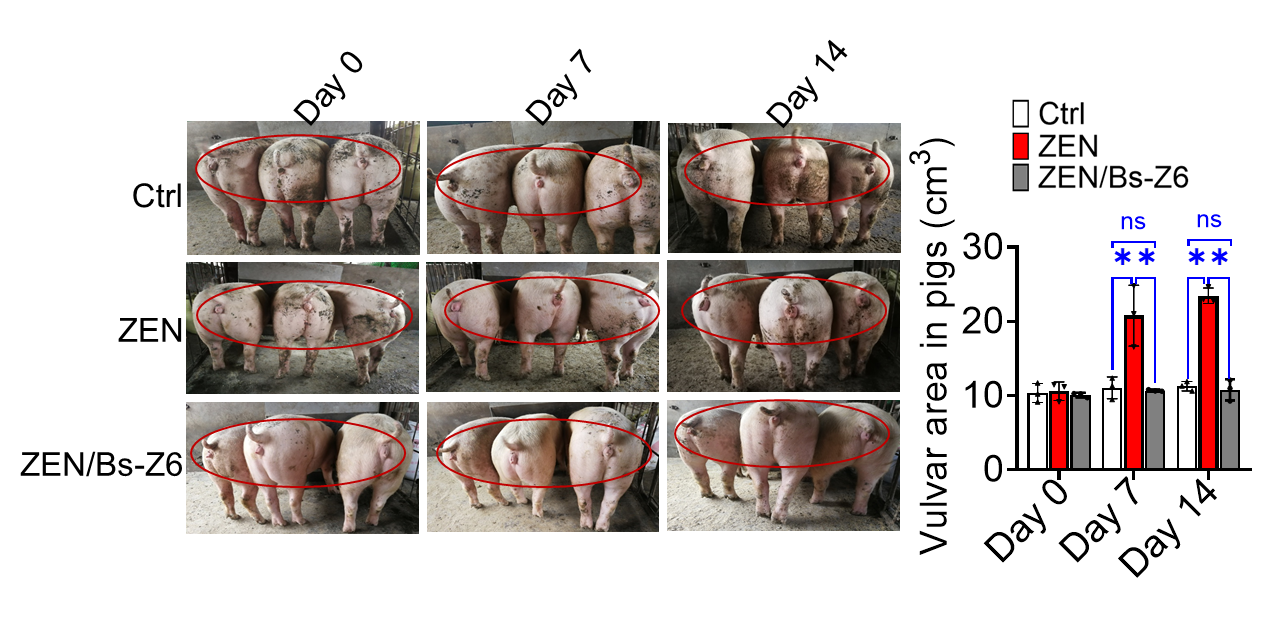
 **Supplemental Fig. S7 (Related to Fig. 7a).** During phase 3, modified microbial metabolites by recombinant Bs-Z6 strain rescue the vulvar area of pigs that expose to ZEN (n=4). Bar values are means ± SEM. ******P* < 0.05.
