# Supplementary material for: Zearalenone disturbs the reproductive-immune axis in pigs: the role of gut microbial metabolites
Source: Microbiome. 2022 Dec 19;10:234. doi: 10.1186/s40168-022-01397-7 (PMC9762105; doi:10.1186/s40168-022-01397-7)
Supplement: Supplementary file 14 — Additional file 13: Supplemental Fig. S8. (Related to Fig. 7e-j). During phase 3, modified microbial metabolites by recombinant Bs-Z6 strain corrected gene expression patterns and functions of reproductive organs (e.g., uterus) and immune organs (e.g., thymus) in pigs that exposed to ZEN (n=4). [file 40168_2022_1397_MOESM13_ESM.docx]

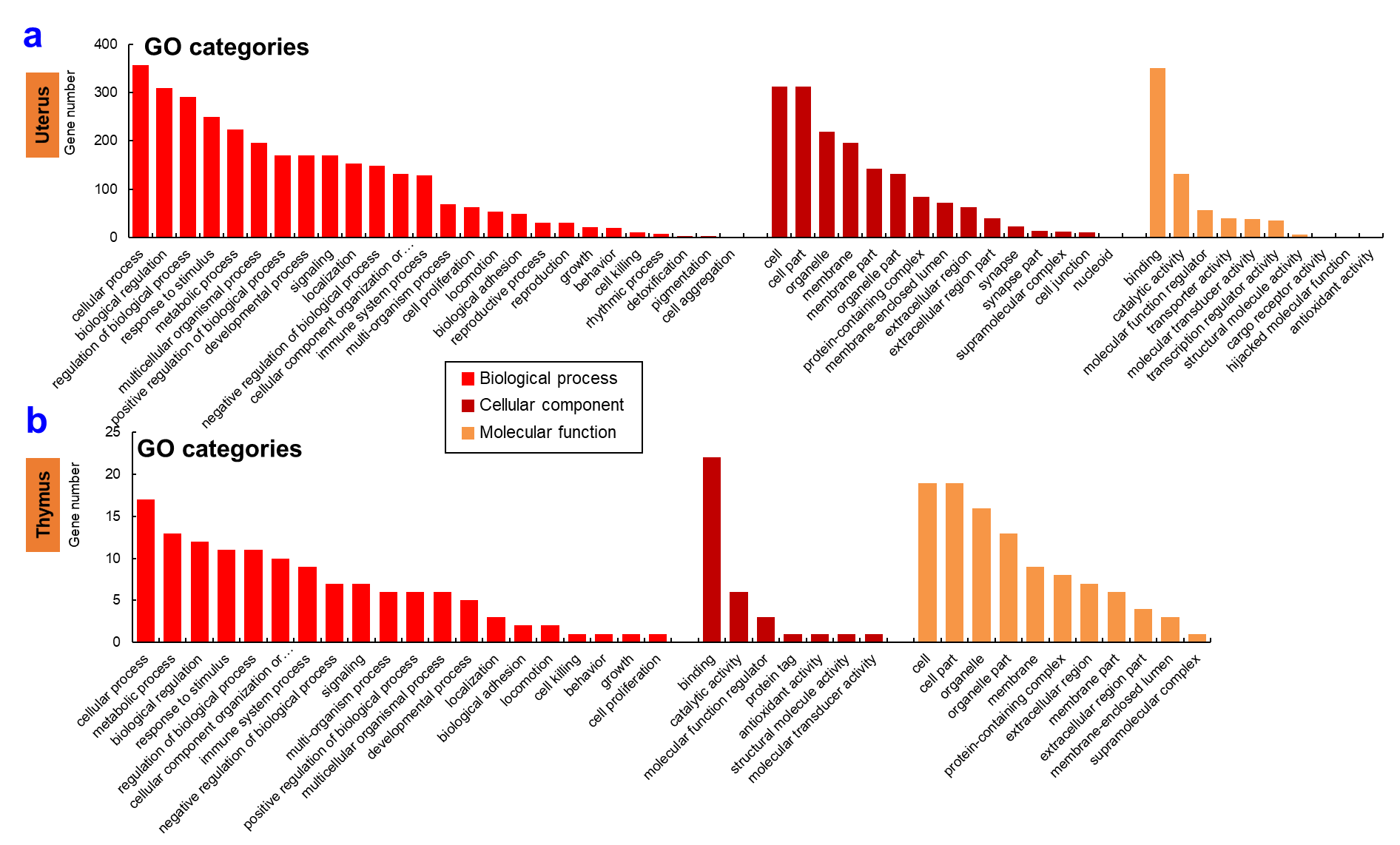

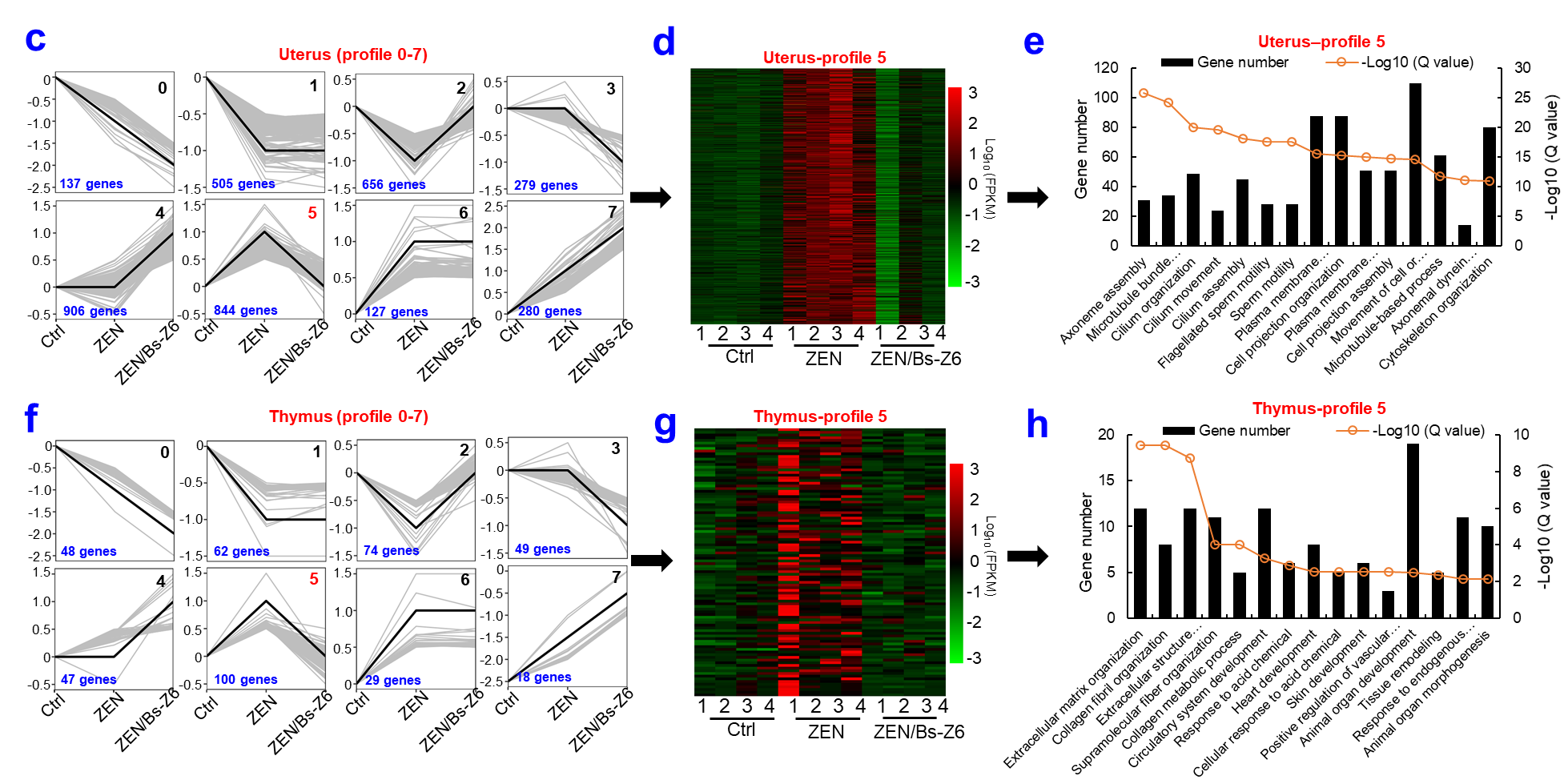

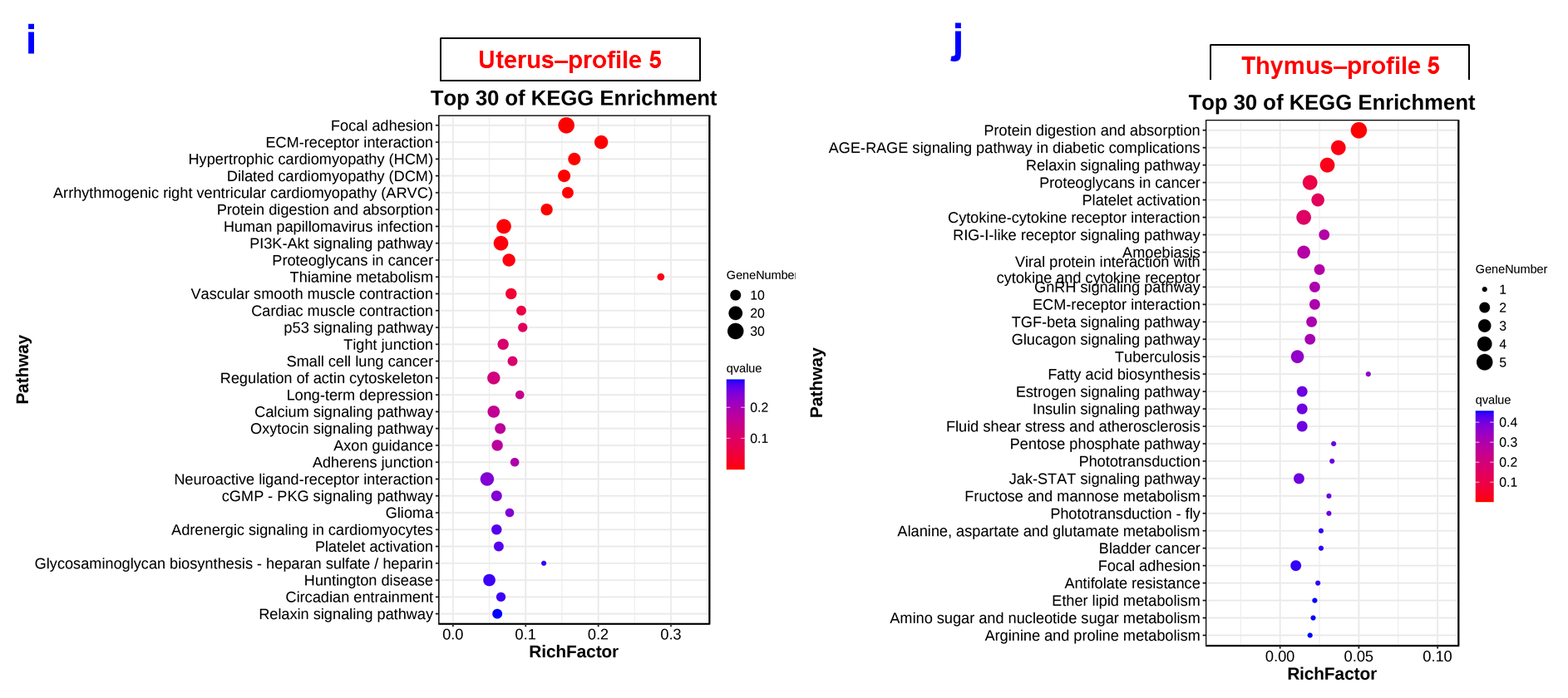


**Supplemental Fig. S8 (Related to Fig. 7e-j).** During phase 3, modified microbial metabolites by recombinant Bs-Z6 strain corrected gene expression patterns and functions of reproductive organs (e.g., uterus) and immune organs (e.g., thymus) in pigs that exposed to ZEN (n=4).

**a, b,** GO classification of genes enriched in profile 2 of reproductive organs (e.g., uterus; **a**) and immune organs (e.g., thymus; **b**). **c**, **f,** Identification of gene expression patterns among the Ctrl, ZEN and ZEN/Bs-Z6 groups in uterus (**c**) and thymus (**f**). **d**, **g,** Heatmap of genes enriched in profile 5 of uterus (**d**) and thymus (**g**). **e**, **h,** Top 15 GO enrichment terms of genes enriched in profile 5 of uterus (**e**) and thymus (**h**). **I**, **j,** Top 30 KEGG pathways of genes enriched in profile 5 of uterus (**i**) and thymus (**j**).
