# Supplementary material for: Zearalenone disturbs the reproductive-immune axis in pigs: the role of gut microbial metabolites
Source: Microbiome. 2022 Dec 19;10:234. doi: 10.1186/s40168-022-01397-7 (PMC9762105; doi:10.1186/s40168-022-01397-7)
Supplement: Supplementary file 15 — Additional file 14: Supplemental Table S6. Key resources table. [file 40168_2022_1397_MOESM14_ESM.docx]

**Supplemental Table S6. Key resources table.**

| REAGENT or RESOURCE | SOURCE | IDENTIFIER |
| --- | --- | --- |
| Antibodies | | |
| Anti-CD63 | Santa Cruz | Cat#SC-5275 |
| Anti-HM74A (GPR109A | Santa Cruz | Cat#SC-377292 |
| Anti-Phospho-NF-κB p65 (Ser536) | Cell signaling | Cat#3033S |
| Anti-NF-κB p65 | Cell signaling | Cat#8242S |
| Anti-E-cadherin (24E10) | Cell signaling | Cat#3195P |
| Anti-GAPDH (14C10) | Cell signaling | Cat#2118 |
| Anti-Phospho-p44/42 MAPK (Erk1/2) (Thr202/Tyr204) | Cell signaling | Cat#9101S |
| Anti-p44/42 MAPK (Erk1/2) | Cell signaling | Cat#9102S |
| Anti-Phospho-p38 MAPK (Thr180/Tyr182) | Cell signaling | Cat#4511S |
| Anti-p38 MAPK | Cell signaling | Cat#9212S |
| α-Tubulin (11H10) | Cell signaling | Cat#2125S |
| Anti-TLR4 | Abcam | Cat#ab13556 |
| Anti-GPR41 | Affinity | Cat#AF9075 |
| Anti-Occludin | Proteintech | Cat# 27260-1-AP |
| Anti-CD544/ICAM-1 | Beyotime | Cat#AF0195 |
| Anti-Claudin-1 | Beyotime | Cat#AF6504 |
| Anti-ZO-1 | Beyotime | Cat#AF8394 |
| Chemicals, peptides, and recombinant proteins | | |
| Zearalenone | Pribolab | STD#4012 |
| Zearalanone | Pribolab | STD#4025 |
| α-Zearalenol | Pribolab | STD#4023 |
| β-Zearalenol | Pribolab | STD#4024 |
| α-Zeranol | Pribolab | STD#4026 |
| β-Zeranol | Pribolab | STD#4027 |
| 13C_18_-Zearalenone | Pribolab | STD#4012U |
| 13C_18_-Zearalanone | Pribolab | STD#4025U |
| 13C_18_-α-Zearalenol | Pribolab | STD#4023U |
| 13C_18_-β-Zearalenol | Pribolab | STD#4024U |
| 13C_18_-α-Zeranol | Pribolab | STD#4026U |
| 13C_18_β-Zeranol | Pribolab | STD#4027U |
| Immunoaffinity Column for the Purification Zeranols | Pribolab | PriboFast® IAC-201-3 |
| TRIzol Reagent | Thermo Fisher Scientific | Cat# 15596-026 |
| Paraformaldehyde | Sigma Aldrich | Cat# P6418 |
| Tween20 | Solarbio | Cat# T8220 |
| Nonidet P-40 | Solarbio | Cat# N8030 |
| TritonX-100 | Solarbio | Cat# T8200 |
| LE-Agrose | Sigma Aldrich | Cat# 9012-36-6 |
| Sodium butyrate | Selleck | Cat#s1999 |
| SDS | Sigma Aldrich | Cat# 151-21-3 |
| Glycine | Solarbio | Cat# G8200 |
| Tris | Solarbio | Cat# T8060 |
| RIPA protein lysis buffer | Beyotime, P0013B | Cat# P0013B |
| Goat anti-Rabbit IgG (H+L) Fluor594-conjugated | Affinity | Cat#S0006 |
| Reactive Oxygen Species Assay Kit | Beyotime | Cat#S0033S |
| ECOR I | New England Biolabs | Cat#R3101 |
| BamH I | New England Biolabs | Cat#R0136S |
| Proteinase inhibitor cocktails | Beyotime | Cat# P1010 |
| TNF-α ELISA kit | Sigma Aldrich | Cat# RAB0478-1KT |
| IFN-γ ELISA kit | Nanjing Jiancheng Bioengineering Institute | Cat# H025 |
| IL-1α ELISA kit | Nanjing Jiancheng Bioengineering Institute | Cat# H001 |
| IL-1β ELISA kit | Nanjing Jiancheng Bioengineering Institute | Cat# H002 |
| IL-6 ELISA kit | R&D Systems | Cat# M6000B |
| IL-12 ELISA kit | Nanjing Jiancheng Bioengineering Institute | Cat# H010 |
| SsoFast EvaGreen Supermix | Bio-Rad | Cat# 1725201 |
| Catalase (CAT) assay kit | Nanjing Jiancheng Bioengineering Institute | Cat# A007-1-1 |
| Penicillin/streptomycin | Thermo Fisher Scientific | Cat#15140122 |
| DMEM/F12 medium | Thermo Fisher Scientific | Cat# 11320033 |
| Pig's diet for phase 1 | Shenzhen Premix Inve Nutrition Co.,Ltd | N/A |
| Pig's diet for phase 2 | Shenzhen Premix Inve Nutrition Co.,Ltd | N/A |
| Pig's diet for phase 3 | Shenzhen Premix Inve Nutrition Co.,Ltd | N/A |
| FBS (fetal bovine serum) | Gibco | Cat#10099-141C |
| Experimental models: Organisms/strains | | |
| Pigs in phase 1 | Shenzhen Premix Inve Nutrition Co.,Ltd | N/A |
| Pigs in phase 2 | Shenzhen Premix Inve Nutrition Co.,Ltd | N/A |
| Pigs in phase 3 | Shenzhen Premix Inve Nutrition Co.,Ltd | N/A |
| IPEC-J2 (cell line) | Institute of Animal Nutrition, Sichuan Agricultural University | N/A |
| *Bacillus subtilis* 168 | This paper | N/A |
| E. coli cells DH5a | Invitrogen | Cat#10361012 |
| Recombinant pWBZ1 Plasmid | This paper | N/A |
| Recombinant pWBZ27 plasmid | This paper | N/A |
| Oligonucleotides | | |
| RT-qPCR primers, see suppl Table S.. | This paper | N/A |
| Deposited data |  |  |
| RNA sequencing data | This paper | Update later on |
| 16S rRNA sequencing data | This paper | Update later on |
| Software and algorithms | | |
| IBM SPSS Statistics 23 (SPSS Inc., Chicago, IL) | IBM | N/A |
| GraphPad 8.0 PRISM® | GraphPad Software | [https://www.graphpad.com](https://www.graphpad.com/) |
| Adobe Illustrator | Adobe Illustrator | https://www.adobe.com/products |
| Biorender | Biorender | https//biorender.com/ |
