# Supplementary material for: Zearalenone disturbs the reproductive-immune axis in pigs: the role of gut microbial metabolites
Source: Microbiome. 2022 Dec 19;10:234. doi: 10.1186/s40168-022-01397-7 (PMC9762105; doi:10.1186/s40168-022-01397-7)
Supplement: Supplementary file 16 — Additional file 15: Supplemental Table S7. List of primers used in RT-qPCR. [file 40168_2022_1397_MOESM15_ESM.docx]

**Supplemental Table S7. List of primers used in RT-qPCR.**

| Genes | Forward primer sequence (5’ -> 3’) | Reverse primer sequence (5’ -> 3’) |
| --- | --- | --- |
| Oxidative stress markers | | |
| *CuZn-sod* | GAGACCTGGGCAATGTGACT | CTGCCCAAGTCATCTGGTTT |
| *Mn-sod* | TGGAGGCCACATCAATCATA | AGCGGTCAACTTCTCCTTGA |
| *e-sod* | ACGCTGCTCTGTGCTTACCT | TCAACTCCTGCCAGATCTCC |
| *PGC1a* | GATGTGTCGCCTTCTTGTTC | CATCCTTTGGGGTCTTTGAG |
| *GSH-Px* | CAAGTCCTTCTACGACCTCA | GAAGCCAAGAACCACCAG |
| *CAT* | AACTGTCCCTTCCGTGCTA | CCTGGGTGACATTATCTTCG |
| Pro-inflammatory cytokines | | |
| *TNF-α* | TGGCCCCTTGAGCATCA | CGGGCTTATCTGAGGTTTGAGA |
| *IFN-γ* | GAGCCAAATTGTCTCCTTCTAC | CGAAGTCATTCAGTTTCCCAG |
| *IL-1α* | CAGCCAACGGGAAGATTCTG | AATGGCTTCCAGGTCGTCAT |
| *IL-1β* | TGATGCCAACGTGCAGTCTA | GGAGAGCCTTCAGCATGTGT |
| *IL-6* | GCTATGAACTCCCTCTCCACA | ACCTTTGGCATCTTCTTCCAG |
| *IL-12p35* | CGTGCCTCGGGCAATTATA | CGCAGGTGAGGTCGCTAGTT |
| Validation for RNA-seq data | | |
| *ILA* | CGAACCCGTGTTGCTGAAGGAG | TGGATGGGCGGCTGATTTGAAG |
| *FGG* | TGGTTGGTGGATGAACAAGTGT | TGCCACCTGGGTAATAAACTCCAT |
| *CCL-26* | CCCAGCAGGCTGTGATATTCA | CCCATGTTTCCTGCGGTTT |
| *WFDC2* | GACAACCTCAAGTGCTGCAAG | CATTGCGGCAGCATTTCAAC |
| *WFIKKN2* | TCATCATCATGGGCGAGGTG | TTGAGGACGTCGCAGGTTTT |
| *PLAUR* | CAAATGGCTTGCAGTGTTAC | GGCTTCCAGACATTGATTCA |
| *TNFAIP8L3* | GCTCTCCAAGCTCCTACACG | GAGGTTTGGCCTACAGTCCC |
| *IL20RA-1* | GTGTACTGCGTCCTTGTGGA | GAAGTAGCCCGTCACGGAAA |
| *SLPI* | CAAGTGCACAAGTGACTGGC | GGCCATAGACCACTGGACAC |
| Tight junction proteins | | |
| *Occludin* | ATCAACAAAGGCAACTCT | GCAGCAGCCATGTACTCT |
| *Claudin-1* | TACTTTCCTGCTCCTGTC | AAGGCGTTAATGTCA ATC- |
| *ZO-1* | GAGTTTGATAGTGGCGTT | GTGGGAGGATGCTGTTGT |
| House-keeping genes | | |
| *HPRT* | GGACTTGAATCATGTTTGTG | CAGATGTTTCCAAACTCAAC |
| *B2M* | CAAGATAGTTAAGTGGGATCG | TGGTAACATCAATACGATTTC |
| *HMBS* | AGGATGGGCAACTCTACCTG | GATGGTGGCCTGCATAGTCT |
| *GAPDH* | CAGCAATGCCTCCTGTACCA | ACGATGCCGAAGTTGTCATG |
| *β-actin* | CTGGAACGGTGAAGGTGA | TTTGGAAAGGCAGGGACT |
